# Postmovement Beta Synchronization Induced by Speed Effects on IHI from the Ipsilateral to Contralateral Motor Cortex

**DOI:** 10.1523/ENEURO.0370-24.2025

**Published:** 2025-03-20

**Authors:** Xiangzi Zhang, Shengyao Zhang, Haoyuan Zhang, Houmin Wang, Jinyi Long

**Affiliations:** ^1^School of Psychology, Northwest Normal University, Lanzhou, Gansu 730070, China; ^2^College of Basic Medicine, Jinzhou Medical University, Jinzhou, Liaoning 121001, China; ^3^School of Computer Science and Engineering, Guangdong Ocean University, Yangjiang, Guangdong 529500, China; ^4^College of Information Science and Technology, Jinan University, Guangzhou, Guangdong 510632, China

**Keywords:** directed coherence, interhemispheric inhibition, paired-pulse TMS, postmovement beta synchronization

## Abstract

Beta event-related spectral perturbation, including bilateral movement-related beta desynchronization (MRBD) and postmovement beta synchronization (PMBS), can be evoked by unilateral speed movement. A potential correlation might exist between power (de)synchronization and interhemispheric coherence during movement execution. However, during the PMBS phase, the existence of interhemispheric coupling and the effect of speed on it are largely undiscovered. To answer this question, we investigated eight healthy, right-handed volunteers using a combination of electroencephalography, transcranial magnetic stimulation, and electromyography. We explored interhemispheric (directed) coherence during isotonic right index finger abduction movements at two speeds: ballistic and self-paced. We discovered that (1) interhemispheric coherence was greater during the PMBS than during the MRBD period. Furthermore, ballistic movement induced a larger coherence during the PMBS period, but not during the MRBD period. (2) In the MRBD phase, directed coherence from the contralateral motor cortex (CM1) to the ipsilateral motor cortex (IM1) was larger, with a reverse tendency observed during the PMBS period. Additionally, in ballistic movement, directed coherence from IM1 to CM1 was stronger and positively correlated with coherence, with no effect of speed on directed coherence detected in the MRBD phase. To explore the causality of interhemispheric coherence during the PMBS period, we investigated the interhemispheric inhibition (IHI) from IM1 to CM1 at different speeds. A stronger IHI from IM1 to CM1 at PMBS peak time was demonstrated, which was enhanced during ballistic movement. Additionally, IHI was negatively correlated with PMBS, and movement speed was positively associated with interhemispheric coupling during the PMBS period and IHI from IM1 to CM1.

## Significance Statement

The present study explored interhemispheric (directed) coherence during isotonic right index finger abduction movements at two speeds: ballistic and self-paced. We discovered a dominance of interhemispheric coherence during the postmovement beta synchronization (PMBS) period of ballistic movement. Furthermore, directed coherence from the contralateral motor cortex (CM1) to the ipsilateral motor cortex (IM1) was more predominant in the movement-related beta desynchronization phase, with a reverse tendency observed during the PMBS period. Advanced exploration revealed a stronger interhemispheric inhibition (IHI) from IM1 to CM1 at PMBS peak time, which was enhanced during ballistic movement. IHI was negatively correlated with PMBS, and movement speed was positively associated with interhemispheric coupling during the PMBS period and IHI.

## Introduction

Unilateral movement can induce alternation of beta rhythms in bilateral hemispheres (Pfurtscheller and Da Silva, 1999; Qiu et al., 2016). Reflected by electroencephalography (EEG) signals, this phenomenon is represented by a beta event-related spectral perturbation (ERSP; Pfurtscheller and Da Silva, 1999; Qiu et al., 2016). Beta ERSP encompasses a decreased beta power change relative to the baseline, namely, movement-related beta desynchronization (MRBD), followed by an increase in beta power after the termination of movement, known as postmovement beta synchronization (PMBS; Pfurtscheller and Da Silva, 1999; Deiber et al., 2015; Qiu et al., 2016). Additionally, interhemispheric beta coherence between motor areas arises during both unimanual and bimanual rhythmic movements (Mima et al., 2000; Gross et al., 2005), serving as evidence of the functional connections between brain regions (Sklar et al., 1972; Busk and Galbraith, 1975; Deiber et al., 2015).

The association between coherence and MRBD and PMBS over different time courses has been investigated in previous studies. Leocani et al. (1997) reported an increase in coherence over sensorimotor and frontal areas during the MRBD phase. Manganotti et al. (1998) revealed active intercommunication among bilateral, mesial central, and prefrontal regions during the MRBD period. The power increase in coupling during the MRBD period indicates that the cortex is effectively activated and involved in motor control (Leocani et al., 1997; Manganotti et al., 1998). This coupling became more intense as movement complexity increased (Manganotti et al., 1998). However, prior research also indicated inconsistent results regarding the effect of the movement rate on interhemispheric coherence (Serrien and Brown, 2002; Toma et al., 2002). For example, Toma et al. (2002) suggested that motor cortical activation and coupling were greater for faster movements, while Serrien and Brown (2002) found that coupling between the primary sensorimotor cortices in the beta frequency band was reduced with increasing movement speed, and this effect was more pronounced in the antiphase than the in-phase mode. As such, understanding interhemispheric coupling under different velocity conditions during the MRBD period of movement is crucial for comprehending the physiological phenomena and functional significance of the bilateral motor cortex.

Notably, existing studies indicate that beta rhythms over bilateral sensorimotor hand areas or other frontal areas demonstrate a complete lack of bilateral coherence during the PMBS period (Leocani et al., 1997; Gross et al., 2005). Similarly, the bilateral Rolandic mu rhythms show no coupling between the hemispheres during PMBS (Van Leeuwen et al., 1978; Schoppenhorst et al., 1980; Andrew and Pfurtscheller, 1999). The disappearance of coupling corresponds to the cerebral cortex idle hypothesis (Leocani et al., 1997; Gross et al., 2005). However, increasing evidence suggests that the motor cortex does not remain in an idle state during the PMBS period but is involved in motor modulation (Cassim et al., 2001; Gaetz and Cheyne, 2006; He et al., 2020) and is associated with movement parameters (Fry et al., 2016). For instance, greater PMBS has been observed following finger extension movements performed against a heavy load compared with unloaded extensions (Stančák et al., 1997). The increase in the rate of force development is responsible for increments in the amplitude of PMBS (Fry et al., 2016). In other words, faster movement speeds strengthen PMBS (Stančák et al., 1997). Some studies have found that PMBS presence in the contralateral hemisphere or frontal areas is associated with motor inhibition (Gilbertson et al., 2005; Pogosyan et al., 2009; Little et al., 2019; He et al., 2020; Wessel, 2020). PMBS is dependent on motor parameters, which may demonstrate the inhibition mechanism from unilateral movement to the lateral motor cortex. However, several questions remain elusive: (1) the role of the ipsilateral hemisphere in movement control during the PMBS burst; (2) the interaction between hemispheres during the PMBS period, particularly during index finger abduction movements at different speeds and the influence of speed on this interaction; and (3) the specific neuromodulation mechanism manifested by this phenomenon in the sensorimotor cortex.

Aiming to address the questions above, we combined transcranial magnetic stimulation (TMS) and EEG to explore the neural mechanisms of interhemispheric functional connections during the PMBS period. Hemispheric interactions have been studied using TMS pulses applied to the bilateral primary motor cortices (M1; Schulte and Müller-Oehring, 2010). In this paradigm, a conditional stimulus (CS) was applied to M1 in one hemisphere a few milliseconds before the test stimulus (TS) on the opposite side. If the CS affects TMS-induced motor–evoked potential (MEP) amplitude, it can be inferred that the two hemispheres are functionally connected (Rothwell, 2011).

Interhemispheric inhibition (IHI) is crucial in unilateral motion (Mayston et al., 1999; Beaulé et al., 2012). Numerous studies have revealed that IHI increases during unilateral movement (Talelli et al., 2008; Vercauteren et al., 2008; Mooney et al., 2018). It has also been found that inhibition from ipsilateral to contralateral hemispheres decreases during unilateral movement execution (Chen et al., 2003; Tazoe and Perez, 2013). However, due to scarce explorations, IHI from the ipsilateral to the contralateral hemisphere during the PMBS burst after unilateral movement remains unclear. Furthermore, it has been reported that ipsilateral to contralateral IHI can help control the time course of muscle activation and the motor learning process, leading to changes in motor response speed (Meyer and Voss, 2000; Davare et al., 2007). Therefore, understanding how the ipsilateral hemisphere regulates IHI during the PMBS burst at different speeds will help elucidate the functional significance and neural mechanisms of PMBS.

In the present study, we hypothesize that (1) during the MRBD and PMBS periods, the influence of index finger abduction at different speeds on interhemispheric interaction varies and (2) the interhemispheric interaction regulated by speed and PMBS amplitude is associated with the IHI mechanism from the ipsilateral to the contralateral hemisphere.

## Materials and Methods

### Subjects

Eight healthy, right-handed volunteers (three females, 23.2 ± 0.47 years old) were investigated in the present study. The present study is a preliminary, small-sample investigation. The sample size was determined based on previous studies employing similar neurophysiological techniques (Stancák and Pfurtscheller, 1996; Leocani et al., 1997; Manganotti et al., 1998; Mima et al., 2000; Serrien and Brown, 2002; Toma et al., 2002; Szurhaj et al., 2003; Gross et al., 2005; Yao et al., 2007; Stevenson et al., 2011; Qiu et al., 2016; Little et al., 2019). The mean number of participants in these studies was ∼7.33. We initially recruited 10 volunteers; however, 2 withdrew due to personal circumstances. Ultimately, eight subjects completed the experiment, which exceeds the average sample size of the aforementioned studies. Additionally, a power analysis using G*Power 3.1.9.7 was conducted to determine the adequacy of our sample size. The go/no-go paradigm employed in the present study is a well-established experimental paradigm. Its robustness has been repeatedly tested in previous research conducted by our group, with relatively large effect sizes consistently observed (Zhang et al., 2020, 2024). Therefore, based on previous research (Li et al., 2022; Himmelmeier and Werheid, 2024), the effect size was set to *f* = 0.4 and α = 0.05 for the statistical power calculation (Cohen, 2016). The analysis yielded a statistical power of 0.68, which is considered acceptable for neuroelectrophysiological studies (Faul et al., 2007; Häger et al., 2021). All participants were confirmed to be in good physical and mental health, with no dyskinesia or mental illness. Handedness was assessed using the Edinburgh Handedness Inventory (Oldfield, 1971). Written informed consent was obtained from all participants prior to the recordings. The experimental procedures were approved by the local ethics committee at Jinan University and were conducted in accordance with the guidelines established in the Declaration of Helsinki.

### EEG and EMG recordings

EEG data were acquired from 64 scalp sites (extended 10–20 system) using a cap with active Ag/AgCl electrodes (Quickcap64). Wet electrodes were used in the cap, and electrode impedance was maintained at <5 KΩ. The reference electrode was placed on the bilateral mastoid. A Neuroscan-Synamps2 amplifier was used to amplify the EEG signal. The EEG signal was sampled at 1 kHz. A bandpass filter ranging from 0.5 to 40 Hz was applied, along with a 50 Hz notch filter. During off-line data preprocessing, independent component analysis (ICA) using the Infomax algorithm from the EEGlab toolbox (Delorme and Makeig, 2004) was performed. Following visual inspection of scalp maps and time course activations for each ICA component, those clearly associated with eyeblinks were eliminated. The remaining subset of components was back-projected onto the EEG data for subsequent analysis.

The first dorsal interosseous (FDI) muscle was selected for index finger isometric abduction tasks (Yao et al., 2007). Surface electromyography (EMG) signals were recorded bilaterally from the FDI using Ag–AgCl electrodes (10 mm diameter). The EMG signals were amplified and filtered with a bandwidth of 5–2 kHz using a bioamplifier (Neurolog System, Digitimer). The signals were digitized at a rate of 5 kHz using an A/D converter [CED Micro 1401, Cambridge Electronic Design (CED)] and stored on a laboratory computer. Online visual display and off-line analysis were conducted using a custom data collection and conditional averaging software (Spike2 for Windows, Version 9.11, CED). The marks generated by the presentation software (E-Prime 3.0) were transmitted to the EEG acquisition software (Curry 8) for synchronization purposes. Simultaneously, the EMG and TMS equipment acquisition software (Spike2) operated to ensure precise timing and synchronization during the experiment.

### Experimental paradigm

The subjects were seated comfortably in an armchair with both arms flexed at the elbow joint at 90° and supported by a table. During the experiment, the left hand was kept relaxed and rested on the table with the wrist in pronation.

**Figure 1.**
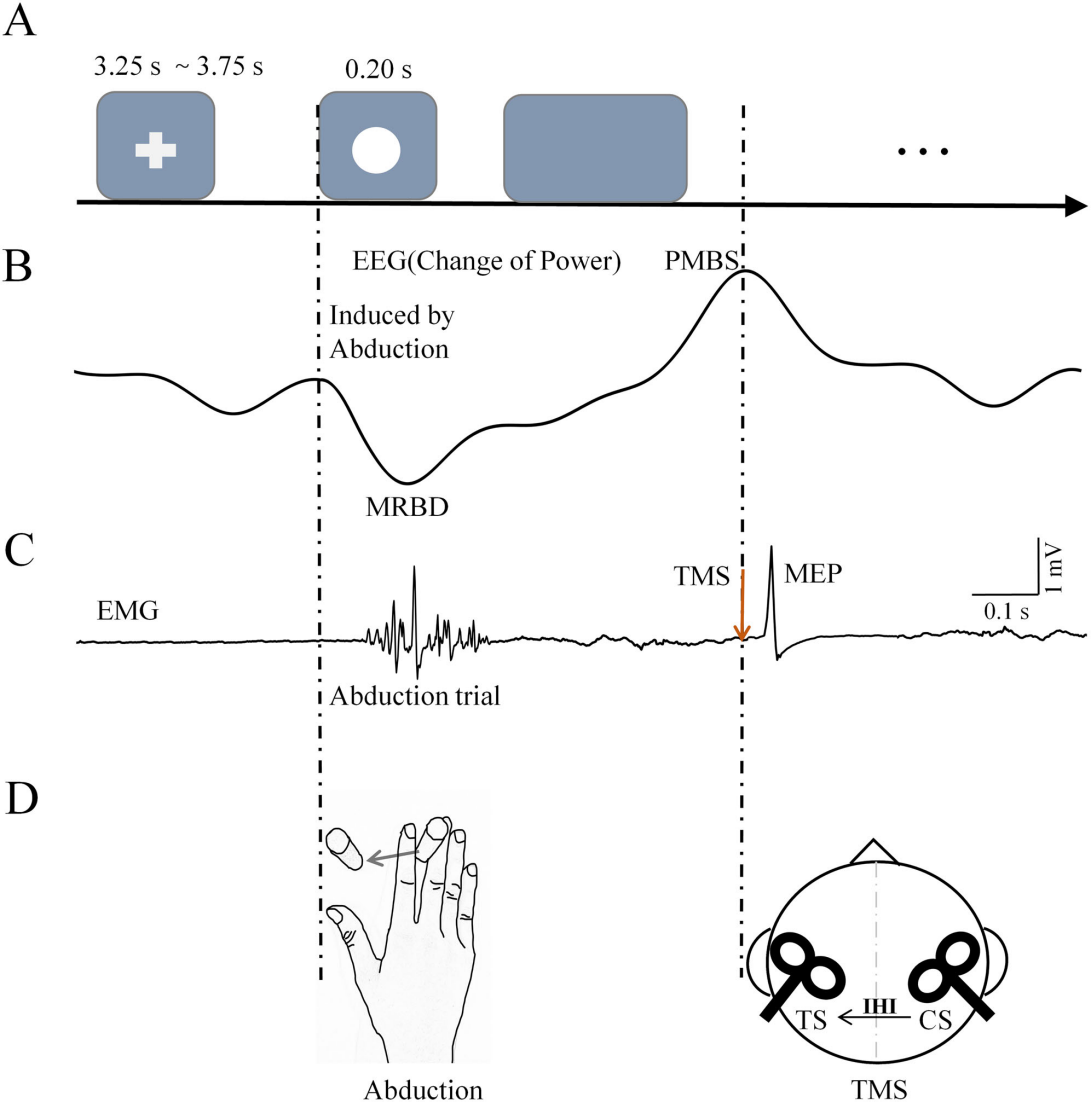
Experimental procedure. ***A***, A schematic diagram of the single experiment task flow. Subjects were instructed to perform index finger abduction movements under different conditions while the white circle was appearing. ***B***, The beta waveforms, which include two stages: MRBD during the execution of the motion and PMBS after movement while performing the task. ***C***, The EMG sequence and MEP from a single task. ***D***, A schematic diagram of the index finger abduction movement and the brain areas stimulated by TMS. MEP, motor-evoked potential; TMS, transcranial magnetic stimulation; MRBD, movement-related beta desynchronization; PMBS, postmovement beta synchronization.

The configuration of the experimental procedure was adapted from previous studies (Ferbert et al., 1992; Yao et al., 2007; Stevenson et al., 2011; Fry et al., 2016; Qiu et al., 2016) and is presented in Figure 1*A*. A fixation cross and a white circle appeared alternately on the screen. At the beginning of each trial, a fixation cross appeared in the center of the screen for a variable delay (3.25–3.75 s). Subjects remained relaxed during this period. Then, an imperative stimulus consisting of a white circle appeared for 0.20 s to denote the performance of an isometric abduction movement with the right index finger. There were two speeds for the isometric abduction movement: ballistic movement and self-paced movement. PMBS could be observed after the isometric abduction movement (Fig. 1*B*). When PMBS peaked, TMS pulses were applied to the primary sensorimotor areas of the left and right hemispheres. The position of the tested right hand is shown in Figure 1*D*. Pegs were used to limit the movement range of the index finger. The distance between the two pegs was adjusted for each subject's comfort. Once determined, the peg distance remained fixed throughout the experiment to ensure equidistant index finger abduction movement. Each speed condition included three blocks, each encompassing 20 trials. The number of trials employed in the present study is consistent with those used in previous research (Ferbert et al., 1992; Fry et al., 2016). Furthermore, we calculated the time–frequency representation (TFR), which has been demonstrated to exhibit a superior signal-to-noise ratio compared with event-related potentials (Baker et al., 1997; Morales and Bowers, 2022).

Additionally, another experiment was performed for comparison. The experimental paradigm was similar to the previous one, with the only difference being the timing of the TMS pulses. In this experiment, TMS pulses were triggered as the PMBS returned to baseline, rather than during the PMBS peak. Prior to the experiments, we determined the time points when PMBS peaked and returned to the baseline. Hence, the experiment with isometric abduction movement was exclusively conducted at the beginning (Zhang et al., 2020). There were two speeds matched for ballistic movement and self-paced movement, respectively, in the index finger abduction experiment. Three blocks, each containing 30 trials in randomized order, were tested, with 15 trials for ballistic movement and the remaining trials for self-paced movement. After each trial, subjects were required to report whether they had missed the task or performed the wrong movement. Additionally, we checked the raw data during off-line analysis to identify any missed trials. For further analysis, we discarded the trials where participants either missed or performed incorrectly. If the discarded trials exceeded 20%, the block was recollected. This led to the allowable exclusion of zero to six trials per block per subject; in practice, no block needed to be recollected.

### TMS

Two figure-of-eight-shaped coils (90 mm mean diameter) were connected to two Magstim 200^2^ magnetic stimulators (Magstim) to deliver the CS and TS, respectively. The CS was given over the ipsilateral motor cortex (IM1) and TS over the contralateral motor cortex (CM1). The handles of the coils were pointed backward and 45° away from the midline and then moved in 1 cm steps to identify the position where TMS pulses produced maximal amplitude responses (Fig. 1*D*). The optimal site where stimulation of slight suprathreshold intensity consistently produced the largest MEPs in the contralateral resting FDI muscles was marked with a pen for each hemisphere on the swimming cap-covered scalp. During the test, the TMS coil was held over the head by the operator's hands.

Resting motor threshold (RMT) was defined as the lowest TMS intensity that evoked an MEP amplitude above 50 μV peak-to-peak in the relaxed FDI muscle at least five times out of 10 consecutive stimuli (Rothwell et al., 1999). Additionally, we collected the TMS measurements of IHI in the following four experimental conditions: (1) PMBS induced by ballistic abduction and at peak time (ballistic, peak); (2) PMBS induced by ballistic abduction and at recovering baseline time (ballistic, baseline); (3) PMBS induced by self-paced abduction and at peak time (self-paced, peak); and (4) PMBS induced by self-paced abduction and at recovering baseline time (self-paced, baseline). Each condition consisted of three blocks with 20 trials included each. To prevent fatigue, conditions were separated by a 10 min break.

### IHI

IHI from the right primary motor cortex (IM1) to the left primary motor cortex (CM1) was measured using a double-pulse TMS protocol, as previously described (Ferbert et al., 1992). The Bistim module delivered two magnetic stimuli sequentially to IM1 and CM1 via separate stimulating coils, allowing investigation of the effect of the first stimulus (CS) on the second stimulus (TS). A suprathreshold CS was applied to the optimal scalp position over IM1, followed by a suprathreshold TS delivered to CM1 10 ms later, consistently evoking IHI (Ferbert et al., 1992). The evaluation of IHI was conducted in accordance with the protocol developed by Talelli et al. (2008) and Zhang et al. (2024). The stimulation intensity for both the CS and TS was adjusted to evoke MEPs ranging from 1 to 1.5 mV in the contralateral FDI muscle at rest. The stimulation intensities of the CS and TS were maintained at consistent levels, with panel intensities set at the same value, and the adjustment range was between 110 and 120% of the RMT. Specifically, the TS intensity was set at 115 ± 5% of RMT, while the CS intensity was set at 114 ± 5% of RMT. Furthermore, when adjusting the TS intensity, it was imperative to ensure that in 10 consecutive stimulations, at least five MEP values in the right-hand FDI muscle fell between 1.5 and 2 mV to maintain consistent TS levels across different conditions. In a single-pulse mode, a suprathreshold TS was delivered exclusively to CM1 either at PMBS peak time or during recovery to the baseline. Both single-pulse and paired-pulse modes were randomly administered, with each condition involving 30 single TS MEPs and 30 CS MEPs. IHI was computed by expressing the amplitude of CS MEPs as a percentage of the amplitude of single TS MEPs (CS MEP * 100 / single TS MEP).

### Data processing

#### EEG analysis

EEG was analyzed using the MATLAB-based FieldTrip toolbox (Matlab, 2021b), developed by the Donders Institute for Brain, Cognition, and Behavior (Oostenveld et al., 2011; http://www.ru.nl/neuroimaging/fieldtrip/), and the EEGLAB toolbox (Delorme and Makeig, 2004), developed by the Swartz Center for Computational Neuroscience (https://sccn.ucsd.edu/eeglab/). The response-locked EEG time series (−0.25 s before and 2.5 s after the stimulus) were extracted for artifact screening. Artifacts were identified as EEG signals with amplitudes exceeding M ± 5 SD. Here, M and SD were computed for each time point based on the response-locked EEG time series across all trials. The study specifically focused on channels C3 and C4 of IM1 and CM1 for analysis.

#### TFR

TFR was calculated across trials using a fast Fourier transform (multi)taper approach with short sliding time windows (Percival and Walden, 1993; Osipova et al., 2006). A frequency band from 5 to 40 Hz (in steps of 0.25 Hz) was analyzed, employing an adaptive time window of three cycles for each frequency (Δ*T* = 3 / *f*). The Hanning taper was employed to minimize spectral leakage and control frequency smoothing. Time windows were advanced in steps of 20 ms. EEG power TFR was computed by averaging the squared absolute values of convolutions across trials. The EEG power at each frequency and time point was normalized by subtracting the average power during the baseline period and then dividing it by the average baseline power. The resulting value was then multiplied by 100, thus expressing the event-related EEG power change relative to the baseline. The aforementioned procedure is described in detail by Nakayashiki et al. (2014) as follows:

$$\begin{array}{c} P_{\mathrm{rest}}=\frac{1}{\left|T_{\mathrm{rest}}\right|}\underset{n\in T_{\mathrm{rest}}}{\sum }\;P_{n} \end{array},$$

$$\begin{array}{c} \mathrm{RP}(n)=\frac{P_{n}-P_{\mathrm{rest}}}{P_{\mathrm{rest}}}\times 100 \end{array}.$$

*P*_rest_ represents the mean power spectrum during the rest period (*T*_rest_) and relative power at each time point, and the relative power at each time point [RP(*n*)] was calculated using the instantaneous power spectrum at each time point (*P*_n_).

The baseline was defined as the time window from 0 to 250 ms prior to the onset of the movement task ( Stančák et al., 1997; Fry et al., 2016; Stevenson et al., 2011). Values above 0 indicated that power at that frequency and time point was higher than the average baseline power and vice versa.

#### Event-related power changes

Event-related power changes in the beta band (14–30 Hz) were assessed. In both the ballistic and self-paced tasks, the duration of MRBD and PMBS typically exceeds 0.5 s (Stancák and Pfurtscheller, 1996). To visualize alterations over different time courses during the abduction tasks, we reduced the dimension to the time domain by averaging the values of beta rhythms in the time–frequency domain (as shown in Fig. 4).

The value of PMBS was defined as the average normalized power over a 200 ms window centered on the peak of the power change. Conversely, the MRBD was defined as the average normalized power over a 200 ms window centered on the trough of the power change during movement. The onset of MRBD was marked by the time at which beta power declined by 10% compared with the baseline (Zhang et al., 2008). Similarly, the end time of MRBD was defined as the point at which beta power returned to 10% below the baseline. The latency of PMBS was identified as the time at which beta power increased by >10% above the baseline, while the termination of PMBS was marked by the return of beta power to 10% above the baseline. The peak time of PMBS was defined as the time point with the maximum beta power during the PMBS burst. The movement duration was constrained within the interval between the initiation and termination of MRBD/PMBS.

It is acknowledged that bilateral PMBS appears at a fixed time in the motor cortex during index finger abduction tasks (Pfurtscheller and Neuper, 1997). The peak and recovery baseline times of PMBS can be calculated for bilateral M1. In the present study, we calculated the average PMBS peak and recovery baseline times across all trials in each speed condition when TMS pulses were triggered. Our work primarily focused on the contribution of IM1 during ipsilateral PMBS bursts. Therefore, the peak time of ipsilateral PMBS was selected to trigger TMS pulses, while the PMBS recovery baseline time was set to trigger TMS for the control condition.

#### Coherence between IM1 and CM1

To detect the functional coupling between bilateral M1 in the frequency domain (5–40 Hz), coherence and Granger causality were calculated. This method measures the proportion of the signal within a particular frequency band that maintains a constant phase relationship between two sources, with values ranging from 0 to 1. For EEG–EEG coherence calculation, each trial of 2.75 s was divided into 50-ms-long nonoverlapping sections. The specific procedure was as follows (Baker et al., 1997):

If the FFT derived from signal *i* (*i* = 1,2) over the *l*th section (*l* = 1, …, *L*) is *F*_i,l_(*f*), then the cross-spectrum is as follows:

$$\begin{array}{c} X_{12}(f)=\frac{1}{L}\overset{L}{\underset{l=1}{\sum }}\;F_{1,l}(f)F_{2,l}^{*}(f) \end{array}.$$

Mark * denotes complex conjugate. Both *F* and *X* are, in general, complex numbers. The autospectrum of one of the signals is given by an analogous expression as follows:

$$\begin{array}{c} S_{i}(f)=\frac{1}{L}\overset{L}{\underset{l=1}{\sum }}\;F_{i,l}(f)F_{i,l}^{*}(f). \end{array}$$

The power spectrum is simply |*S*_i_(*f*)|^2^, and the coherence is given as follows:

$$\begin{array}{c} C_{12}(f)=\frac{{\left|X_{12}(f)\right|}^{2}}{S_{1}(f)S_{2}(f)}. \end{array}$$

Before calculating *C*_12_, the cross-spectrum *X*_12_ and autospectra *S*_i_ are smoothed using a Hanning window in which each point is replaced with the weighted sum of its value and that of the two surrounding points, with weight 0.25, respectively (Farmer et al., 1993). This coherence calculation method is undirected and cannot reflect the information flow between Channel 1 and Channel 2 (C3 and C4).

In order to process coherence values across subjects, individual coherence values were initially calculated for each subject using the previously described method. Subsequently, the values were averaged across all subjects in order to obtain group-level coherence estimates.

#### Causality of directed coherence

Directed coherence measures the extent to which one signal can be predicted by the past history of another (Kamiński and Blinowska, 1991). In this case, Dir-Coh can be interpreted as a measure of causality. Let *X*(*t*) be the activity of two EEG signal at time *t*. Estimating directed coherence is to fit a multivariable autoregressive model to the observed signals. Let the following vector:

$$\begin{array}{c} X(t)={\left[\mathrm{EE}G_{1}(t),\mathrm{EE}G_{2}(t)\right]}^{T} \end{array}.$$

The multivariable autoregressive model is constructed as follows:

$$\begin{array}{c} X(t)=\overset{p}{\underset{i=1}{\sum }}\;A(i)X(t-i)+E(t), \end{array}$$

where *A*(*i*) is a 2 * 2 matrix of coefficients describing the causal influence of the signals at lag *i* on the signals at lag zero, *p* is the order of multivariate autoregressive model, and *E*(*t*) is a vector of prediction errors at each time point. We obtain estimates of the coefficient matrices by solving the multivariate Yule–Walker equations by using the Levinson, Wiggins, and Robinson algorithm. *p* can be estimated comprehensively by Akaike information criterion and Bayesian information criterion. Transforming this convolution equation to the frequency domain yields, by the following convolution theorem:

$$\begin{array}{c} A(f)X(f)=E(f). \end{array}$$

Therefore,

$$\begin{array}{c} X(f)=A{\left(f\right)}^{-1}E(f)=H(f)E(f), \end{array}$$

where *H*(*f*) is the transfer function of the system. |*H*_ij_(*f*)|^2^ is the directional transfer function, representing the causal influence of signal *j* on *i*. Directed coherence is defined as follows:

$$\begin{array}{c} \mathrm{Dir}\;\mathrm{Co}h_{i\leftarrow j}={\left|H_{\mathrm{ij}}(f)\right|}^{2}\frac{S_{\mathrm{jj}}(f)}{S_{\mathrm{ii}}(f)}, \end{array}$$

where *S_ii_*, *S_jj_* are the power spectrum of signal *i *or* j* (*i, j* = 1, 2 in this paper). Directed coherence was estimated for each subject in each condition at frequency bands between 5 and 40 Hz. The order *p* of multivariable autoregressive model in this paper is 10.

The significance level for the directed coherence was calculated as follows (Baker et al., 2006):

$$\begin{array}{c} Z=1-0.05^{\left(\frac{1}{L-1}\right)} \end{array},$$

where *L* is the total number of nonoverlapping sections and the directed coherence was considered significant (*p* < 0.05) if it was greater than *Z*.

To process the directed coherence values across subjects, the individual directed coherence values were initially calculated for each subject using the previously described method. Subsequently, the values were averaged across all subjects in order to obtain group-level estimates.

### Statistical analysis

Repeated-measure two–way ANOVAs were performed to determine the effect of speed (ballistic, self-paced) and hemispheres (CM1, IM1) on EEG parameters (MRBD values, PMBS values, MRBD/PMBS onset time, duration, end time). Repeated-measures two–way ANOVAs were also conducted to study the effect of speed (ballistic, self-paced) and time course (MRBD period, PMBS period) on interhemispheric coherence and interhemispheric directed coherence. Additionally, repeated-measure two–way ANOVAs were performed to examine the effect of speed (ballistic, self-paced) and the time when TMS pulses were triggered (PMBS peak time, PMBS recovery baseline time) on IHI. Bonferroni’s post hoc correction was conducted to detect significant comparisons with a significance level α of 0.05. A priori comparisons were made as specified. Normal distribution was tested by the Shapiro–Wilk test (all *p* > 0.05). The significance level was set at *p* < 0.05, and group data are presented as M ± SD in the text and as SE in the figures.

We explored the correlation among speed, PMBS, coherence, directed coherence, and IHI. The simplest model, a linear model, was chosen to describe the effects of these correlations. This study is an extension of our previous research, which employed a linear model to elucidate the relationships between neural data (Zhang et al., 2020, 2024). In addition to the aforementioned studies, linear models have been demonstrated to offer a robust and straightforward methodology for elucidating correlations and causality in intricate datasets while avoiding the pitfalls of overcomplicated analysis (Gourévitch et al., 2006; Ritchie et al., 2019; Penconek, 2022). Therefore, Pearson's correlation analysis was conducted to evaluate the relationships using IBM SPSS (SPSS) with 1,000 bootstrap samples and a 95% confidence interval.

## Results

### Preexperiment

**Figure 2.**
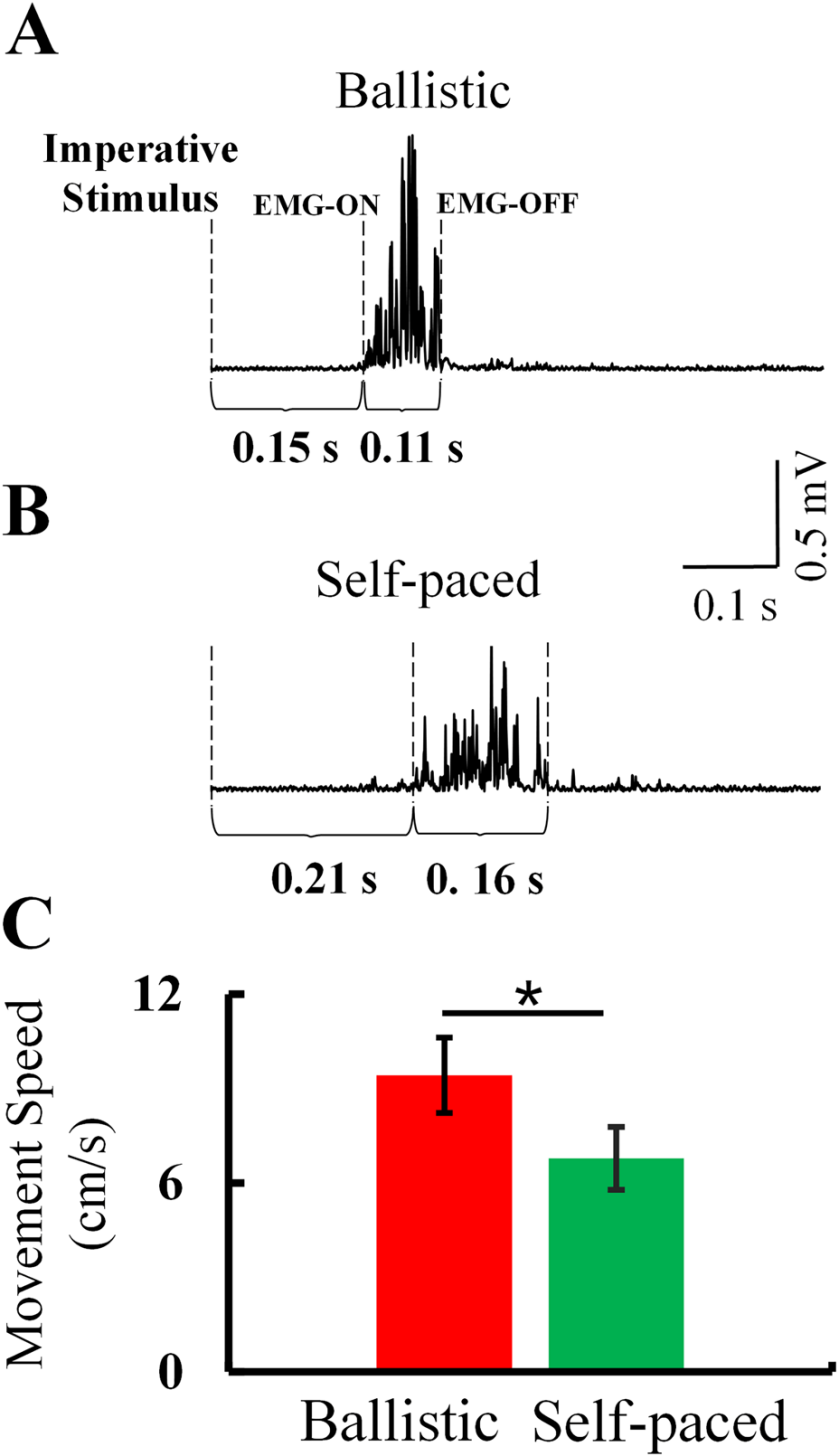
Results of index finger abduction movement at different speeds. ***A***,***B***, EMG sequences after rectification. ***C***, Average movement speeds across subjects during ballistic and self-paced abduction movements. Error bars indicate SEs. *p < 0.05.

**Figure 3.**
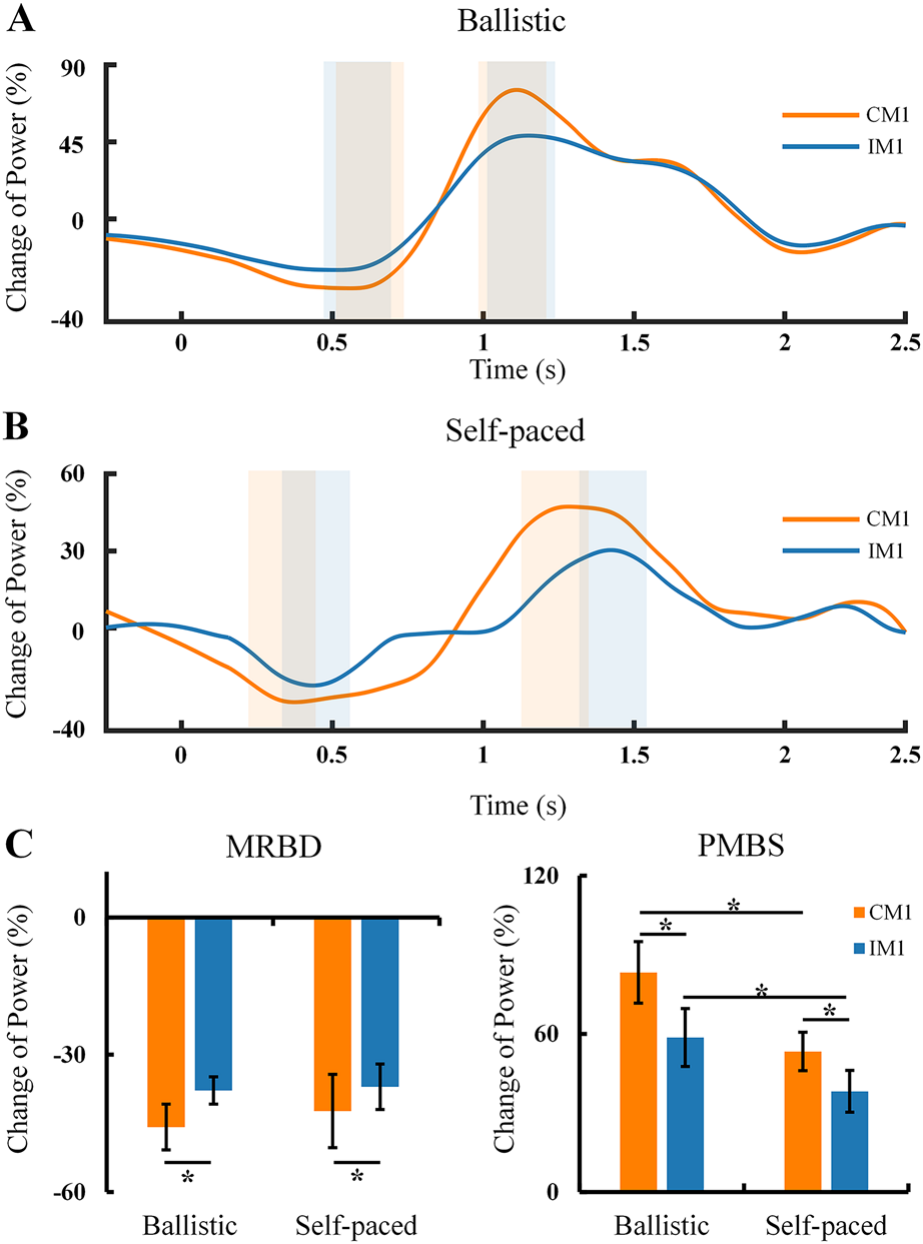
Change in beta rhythm power over the sensorimotor cortex (CM1 and IM1 channels). ***A***,***B***, Power changes in the time domain from a representative subject during ballistic and self-paced abduction movement. ***C***, Average MRBD and PMBS values. The abscissa shows the experimental conditions, and the ordinate shows the average power change across subjects. In panels ***A*** and ***B***, the first two rectangles mark MRBD and the second two rectangles denote PMBS. Error bars indicate SEs. *p < 0.05; MRBD, movement-related beta desynchronization; PMBS, postmovement beta synchronization; IM1, ipsilateral motor cortex; CM1, contralateral motor cortex.

As shown in Figure 2, subjects were required to perform two kinds of abduction at different speeds to evoke beta event-related spectral disturbances (ballistic speed, 8.04 ± 0.93 cm/s; self-paced, 4.26 ± 0.70 cm/s; *p* = 0.002). A decrease in beta power was followed by a rebound synchronization after movement (Fig. 3*A*,*B*). MRBD and PMBS were consistently observed over bilateral hemispheres during abduction movement at varied speeds, with contralateral dominance at both speeds (Fig. 3*A*,*B*).

A significant effect of hemisphere (CM1, IM1) was demonstrated for the MRBD value (Fig. 3*C*; (*F*_(1,31)_ = 12.737; *p* = 0.003). Post hoc analysis indicated that MRBD was larger on CM1 compared with IM1 under the condition of ballistic abduction (CM1, −45.76 ± 5.16%; IM1, −37.83 ± 4.85%; *p* = 0.009) and self-paced abduction (CM1, −42.34 ± 4.16%; IM1, −37.02 ± 4.16%; *p* = 0.020). Additionally, MRBD showed no significant difference between ballistic and self-paced abduction on CM1 (*p* = 0.248) and IM1 (*p* = 0.780).

For PMBS values (Fig. 3*C*), a significant effect of speed (ballistic, self-paced; *F*_(1,31)_ = 21.078; *p* < 0.001) and hemisphere (CM1, IM1; *F*_(1,31)_ = 14.064; *p* = 0.003) was observed. Post hoc analysis revealed that PMBS was larger on CM1 compared with IM1 under ballistic abduction (CM1, 83.30 ± 12.43%; IM1, 58.64 ± 8.82%; *p* = 0.002) and similarly larger under self-paced abduction (CM1, 55.27 ± 9.68%; IM1, 38.24 ± 7.01%; *p* = 0.026). Additionally, PMBS was stronger during ballistic abduction compared with self-paced abduction on both CM1 (*p* < 0.001) and IM1 (*p* = 0.007).

**Figure 4.**
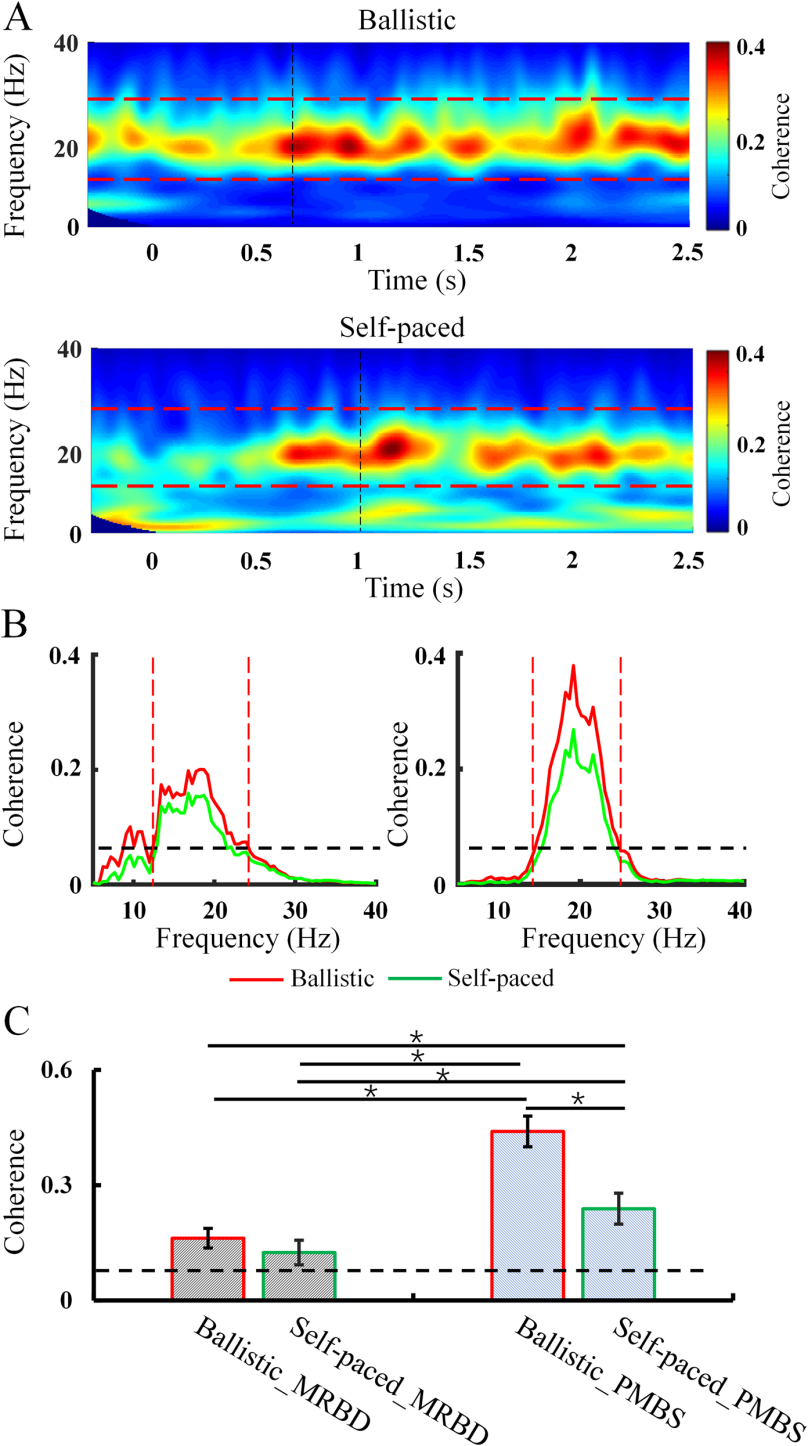
Coherence between CM1 and IM1 at different speeds. ***A***, Coherence in different time–frequency domains from a representative subject (averaged across trials) during speed movement tasks. The dashed lines represent the beta band between 14 and 30 Hz. ***B***, Coherence in the frequency domain during different speed movements. ***C***, Average coherence across all subjects. The abscissa shows the conditions. The ordinate shows the average coherence across subjects. Error bars indicate SEs. *p < 0.05; MRBD, movement-related beta desynchronization; PMBS, postmovement beta synchronization.

As illustrated in Figure 4, the coherence between CM1 and IM1 was quantified. During the baseline period, there was no significant difference in coherence between ballistic and self-paced movements (*F*_(1,14)_ = 0.149; *p* = 0.705). Therefore, baseline analyses were not further pursued. One-sample *t* tests revealed that during the MRBD period, significant beta power above zero was observed in both ballistic (Channel C3, *t* = 4.636; *p* = 0.001; channel C4, *t* = 4.333; *p* = 0.002) and self-paced (channel C3, *t* = 14.454; *p* < 0.001; Channel C4, *t* = 5.096; *p* < 0.001) movements. This verified that there was enough beta power during MRBD period to perform coherence analysis. Significant effects of speed (ballistic, self-paced; *F*_(1,31)_ = 12.030; *p* = 0.005) and time course (MRBD period, PMBS period; *F*_(1,31)_ = 14.485; *p* = 0.003) were observed, as well as a significant effect of their interaction (*F*_(1,31)_ = 7.788; *p* = 0.016; Fig. 4*C*). Post hoc analysis elucidated that coherence was not significantly different between ballistic and self-paced movement during the MRBD period (ballistic, 0.14 ± 0.03; self-paced, 0.12 ± 0.03; *p* = 0.653). However, coherence under the ballistic condition was increased compared with the self-paced condition during the PMBS period (ballistic, 0.43 ± 0.08; self-paced, 0.24 ± 0.07; *p* < 0.001). Coherence was also larger during the PMBS period than the MRBD period under both the ballistic (*p* < 0.001) and self-paced (*p* = 0.032) conditions (Fig. 4*B*,*C*). Although more prominent coherence between bilateral hemispheres was detected during the PMBS period, the direction of information flow between bilateral hemispheres cannot be determined from these results.

**Figure 5.**
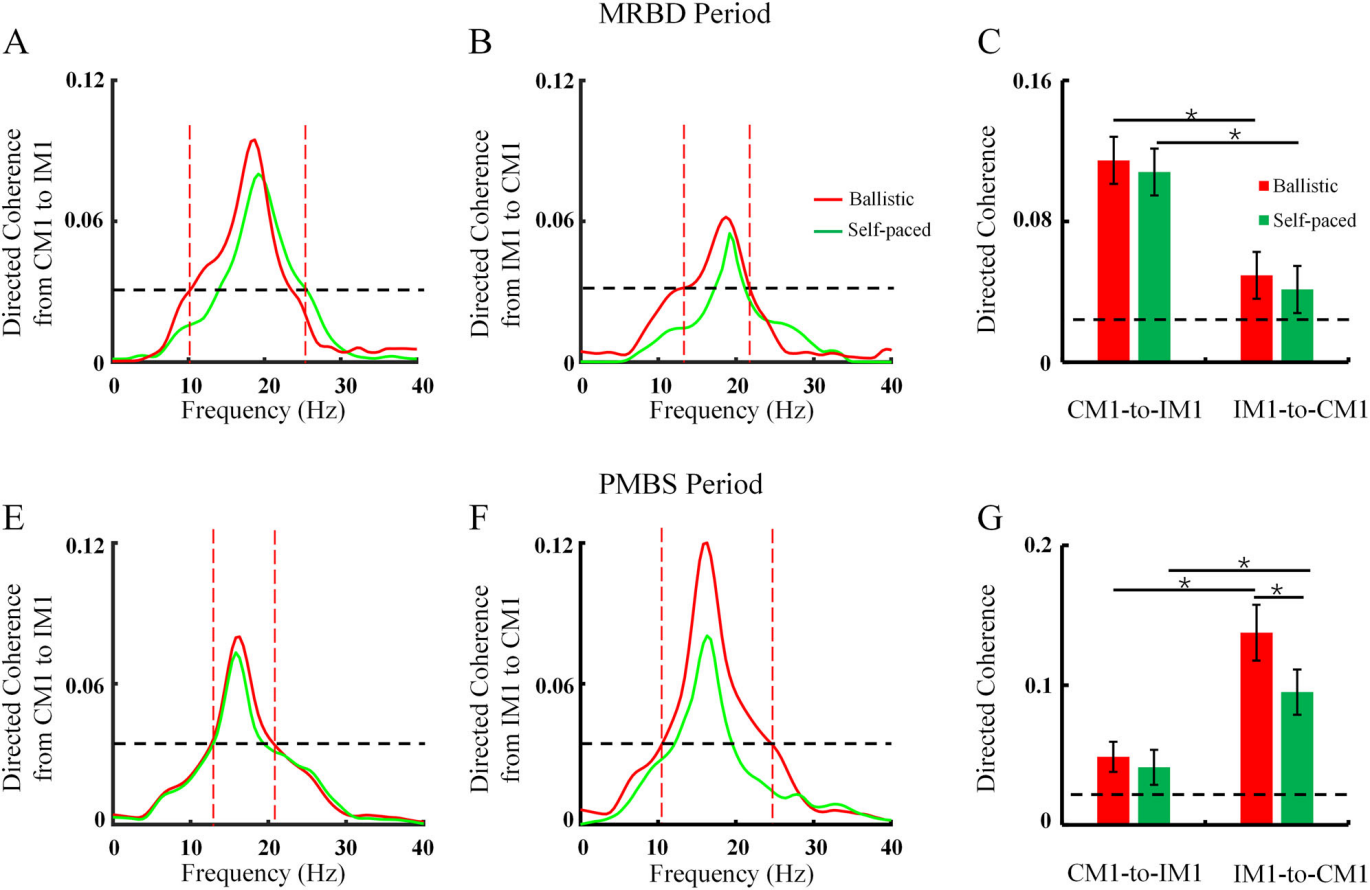
Directed coherence at different time points of MRBD and PMBS. ***A***,***B***, Directed coherence across different frequency domains from a representative subject (averaged across trials) during the MRBD period. ***C***, The average directed coherence during the MRBD period across all subjects. ***E***,***F***, Directed coherence in different frequency domains from a representative subject (averaged across trials) during the PMBS period. ***G***, The average directed coherence in the PMBS period across all subjects. The abscissa indicates the conditions, while the ordinate represents the average coherence across subjects. Dashed lines represent the threshold of directional coherence significance. Error bars denote SEs. *p < 0.05; MRBD, movement-related beta desynchronization; PMBS, postmovement beta synchronization; IM1, ipsilateral motor cortex; CM1, contralateral motor cortex.

Figure 5 presents the results of directed coherence during different time courses of MRND and PMBS, which reflects the causality between the two hemispheres. During the MRBD period, a notable effect of coherence direction (CM1 to IM1, IM1 to CM1) was observed (*F*_(1,31)_ = 11.220; *p* = 0.015; Fig. 5*C*). Post hoc analysis revealed that directed coherence of causality was significantly larger from CM1 to IM1 compared with IM1 to CM1 during the MRBD period induced by both ballistic abduction (CM1 to IM1, 0.087 ± 0.03; IM1 to CM1, 0.037 ± 0.03; *p* = 0.015) and self-paced abduction (CM1 to IM1, 0.081 ± 0.04; IM1 to CM1, 0.033 ± 0.02; *p* = 0.018). However, directed coherence of causality did not significantly differ between ballistic and self-paced movements for either direction (CM1 to IM1, *p* = 0.559; IM1 to CM1, *p* = 0.656). Therefore, it can be concluded that the causal direction of information interaction is predominant from CM1 to IM1 during the MRBD period, with no significant difference between the two speed conditions.

During the PMBS period, the effects of directed coherence (CM1 to IM1, IM1 to CM1) were found to be significant (*F*_(1,31)_ = 53.097; *p* < 0.001), as were the effects of speed (ballistic, self-paced; *F*_(1,31)_ = 7.707; *p* = 0.024; Fig. 5*G*). Post hoc analysis revealed that the causal direction of coherence was greater from IM1 to CM1 compared with CM1 to IM1 during the PMBS period for both ballistic (CM1 to IM1, 0.03 ± 0.03; IM1 to CM1, 0.11 ± 0.04; *p* < 0.001) and self-paced movements (CM1 to IM1, 0.03 ± 0.03; IM1 to CM1, 0.08 ± 0.04; *p* = 0.001). Directed coherence from IM1 to CM1 was significantly increased for ballistic compared with self-paced movements (*p* = 0.004). However, during the PMBS period, the causal direction from CM1 to IM1 did not differ significantly between ballistic and self-paced conditions (*p* = 0.632). Thus, it can be concluded that the causality of information interaction is predominant from IM1 to CM1 during the PMBS period, with a greater effect observed in ballistic than self-paced movement.

Based on the results presented in Figures 4 and 5, coherence during the PMBS period is greater than in the MRBD period, with a stronger causal influence from IM1 to CM1 during PMBS, particularly induced by ballistic movement. This potentially reflects that the functional interaction between the bilateral hemispheres is not independent during the PMBS period, as supported by findings in IHI across the corpus callosum from IM1 to CM1. The results of ANOVAs are presented in Table 1.

**Table 1.** Results of the ANOVAs

Component	Factor	*df1, df2*	*F*	*p*
MRBD value	Speed	1, 31	1.148	0.303
	Hemisphere		12.737	**0**.**003**
	Interaction		0.733	0.410
PMBS values	Speed	1, 31	21.078	**0**.**001**
	Hemisphere		14.064	**0**.**003**
	Interaction		0.733	0.410
Coherence	Speed	1, 31	12.030	**0**.**005**
	Time course		14.485	**0**.**003**
	Interaction		7.788	**0**.**016**
Directed coherence (MRBD period)	Speed	1, 31	0.699	0.435
	Coherence direction		11.220	**0**.**015**
	Interaction		0.00867	0.929
Directed coherence (PMBS period)	Speed	1, 31	7.707	**0**.**024**
	Coherence direction		53.097	**<0.001**
	Interaction		4.219	0.074

### Correlation analysis during MRBD and PMBS periods

**Figure 6.**
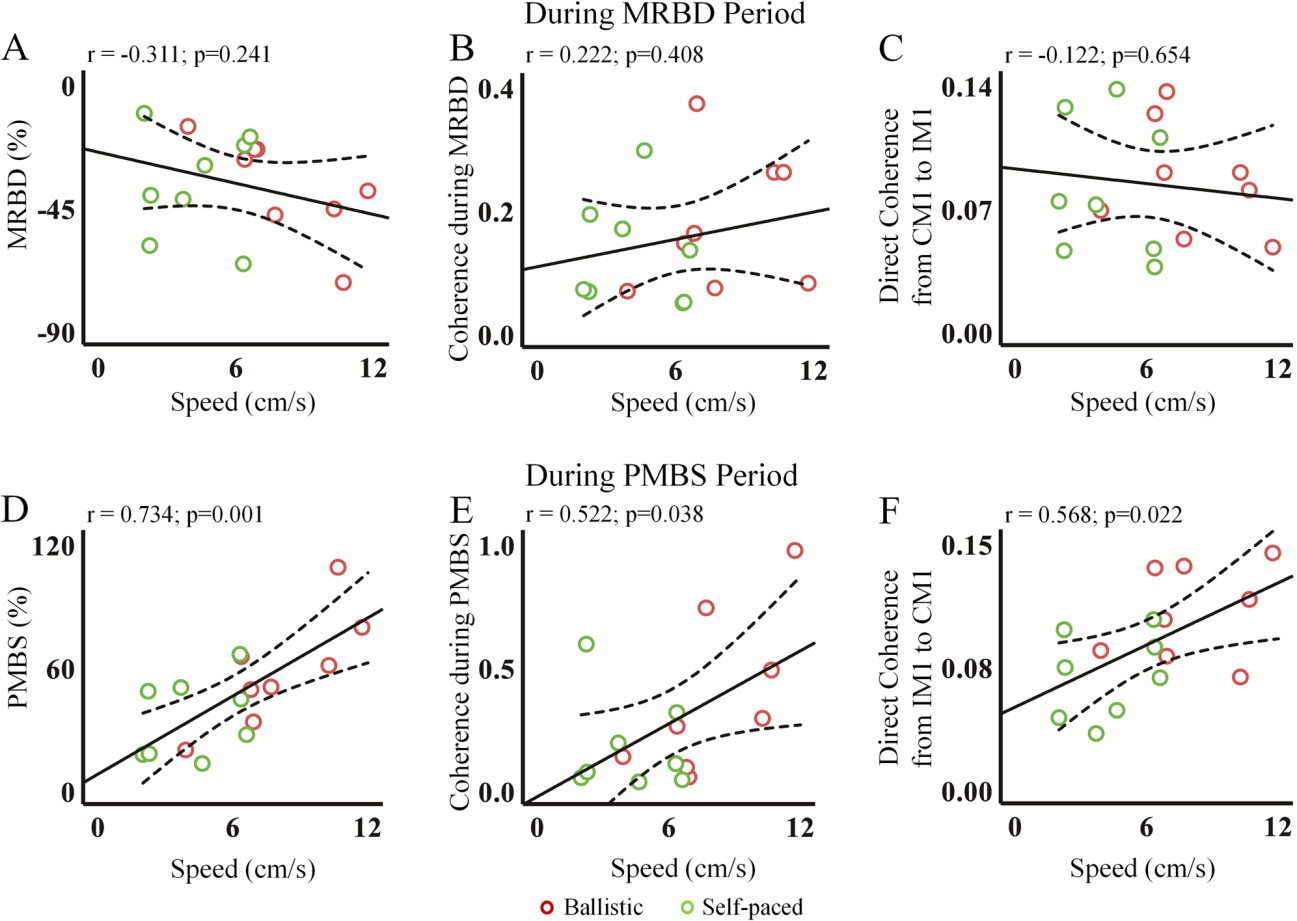
The scatterplots illustrating significant associations. The relationships between speed and EEG parameters are shown. During the MRBD period, no significant correlations were found between speed and MRBD (***A***), coherence (***B***), or directed coherence (***C***). In contrast, during the PMBS period, significant correlations were observed between speed and PMBS (***D***), coherence (***E***), and directed coherence (***F***). The solid and dotted lines represent the regression lines and the 95% confidence limits, respectively. MRBD, movement-related beta desynchronization; PMBS, postmovement beta synchronization.

As illustrated in Figure 6*A–C*, no correlations were identified between speed and MRBD or hemispheric coupling. The directed coherence from CM1 to IM1 was not found to be dependent on speed during the MRBD period. In contrast, Figure 6*D–F* revealed positive correlations between PMBS and speed (*r* = 0.734; *p* = 0.001) and between hemispheric coupling and speed (*r* = 0.522; *p* = 0.038). Additionally, directed coherence from CM1 to IM1 was dependent on speed (*r* = 0.568; *p* = 0.022) during the PMBS period.

**Figure 7.**
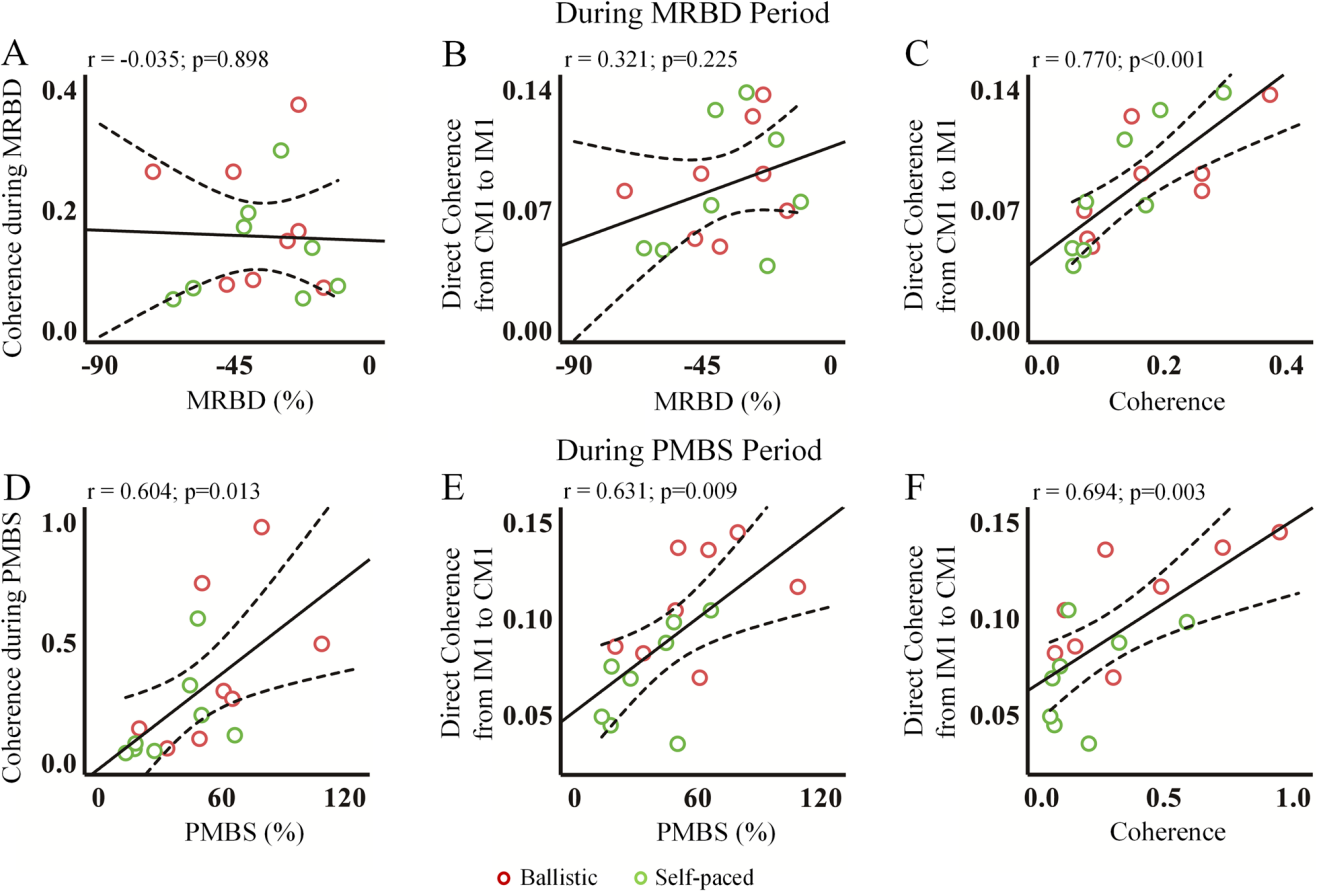
The scatterplots with significant associations. During the MRBD period, insignificant correlations were observed between MRBD and (directed) coherence (***A***,***B***), while a significant correlation between coherence and directed coherence was found (***C***). In the PMBS period, significant correlations were identified between PMBS and (directed) coherence (***D***,***E***), as well as between coherence and directed coherence (***F***). The solid and dotted lines represent the regression lines and the 95% confidence limits, respectively. MRBD, movement-related beta desynchronization; PMBS, postmovement beta synchronization.

As illustrated in Figure 7*A–C*, no correlation was identified between MRBD and coherence or directed coherence during the MRBD period. Conversely, Figure 7*D–F* illustrates a positive correlation between PMBS and coherence (*r* = 0.604; *p* = 0.013) as well as directed coherence (*r* = 0.631; *p* = 0.009) during the PMBS period. Additionally, the correlation between coherence and directed coherence is positive in both MRBD and PMBS periods (MRBD period, *r* = 0.770; *p* < 0.001; PMBS period, *r* = 0.694; *p* = 0.003).

The results of the correlation analysis conducted during the MRBD and PMBS periods are presented in Tables 2 and 3, respectively. Since interhemispheric (directed) coherence was related to PMBS, we further assessed IHI during the PMBS period.

**Table 2.** Results of the correlation during MRBD period

	MRBD period					
	Speed		MRBD		Coherence	
	*r*	*p*	*r*	*p*	*r*	*p*
Directed coherence	−0.122	0.654	0.321	0.225	0.770	**<0.001**
Coherence	0.222	0.408	−0.0035	0.898		
MRBD	−0.311	0.241				

**Table 3.** Results of the correlation during the PMBS period

	PMBS period							
	IHI		Speed		PMBS		Coherence	
	*r*	*p*	*r*	*p*	*r*	*p*	*r*	*p*
Directed coherence	−0.631	**0.009**	0.568	**0**.**022**	0.631	**0**.**009**	0.694	**0.003**
Coherence	−0.321	0.226	0.522	**0**.**0038**	0.604	**0**.**013**		
PMBS	−0.529	**0**.**035**	0.734	**<0**.**001**				
Speed	−0.700	**0**.**003**						

### Results of IHI

**Figure 8.**
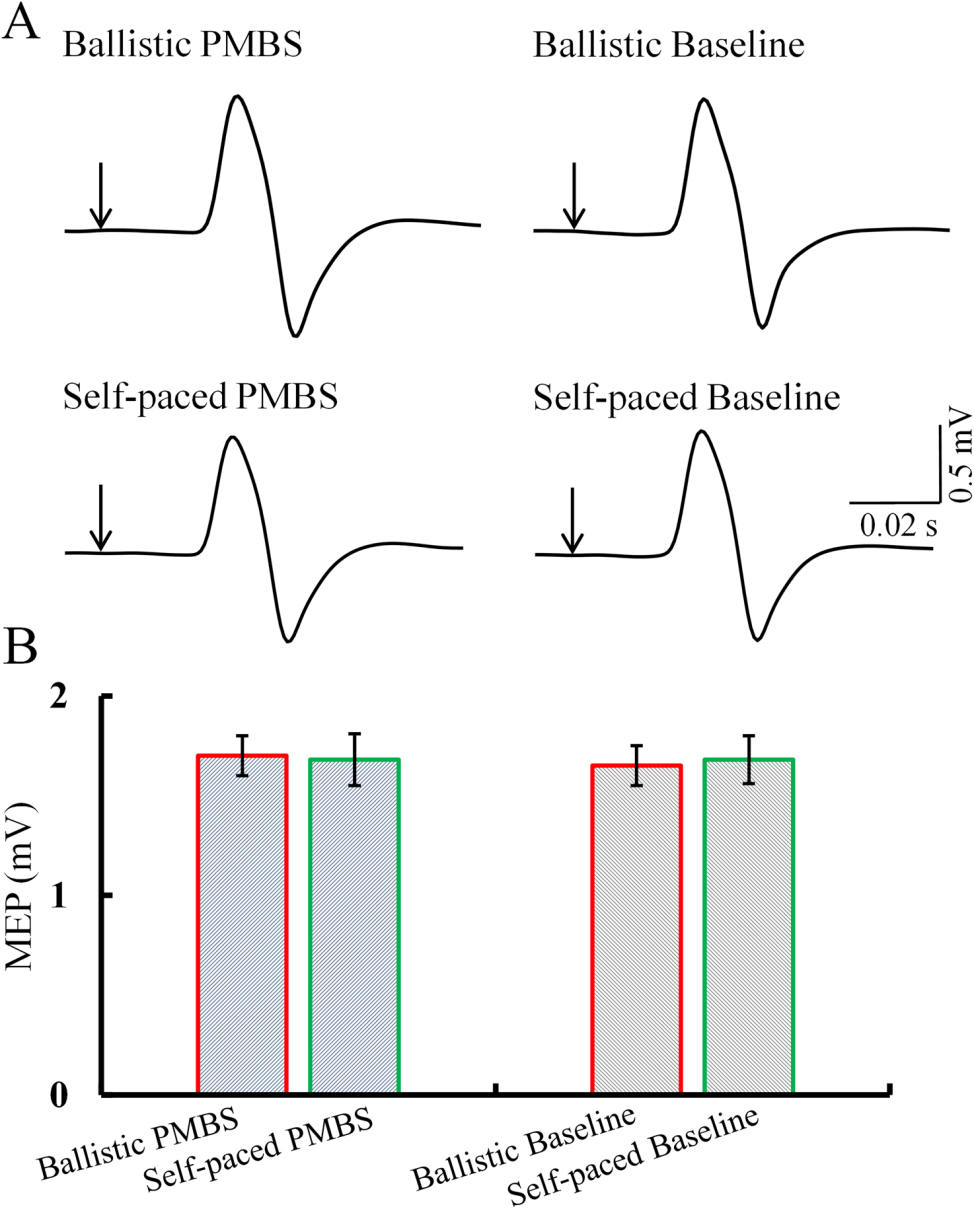
Single-pulse MEP under different conditions. ***A***, Single-pulse MEP from a representative subject (averaged across trials) during various conditions. ***B***, Average of single MEP across subjects. Error bars indicate SEs. *p < 0.05; PMBS, postmovement beta synchronization; MEP, motor-evoked potential.

As illustrated in Figure 8, *A* and *B*, no notable impact of speed (ballistic, self-paced), the timing of TMS pulse initiation (PMBS peak time, PMBS recovery baseline time), or their interaction on TS MEP was discerned. There was no difference in TS MEP across the four conditions. Therefore, correction analysis for TS MEP is unnecessary and was excluded from this paper.

**Figure 9.**
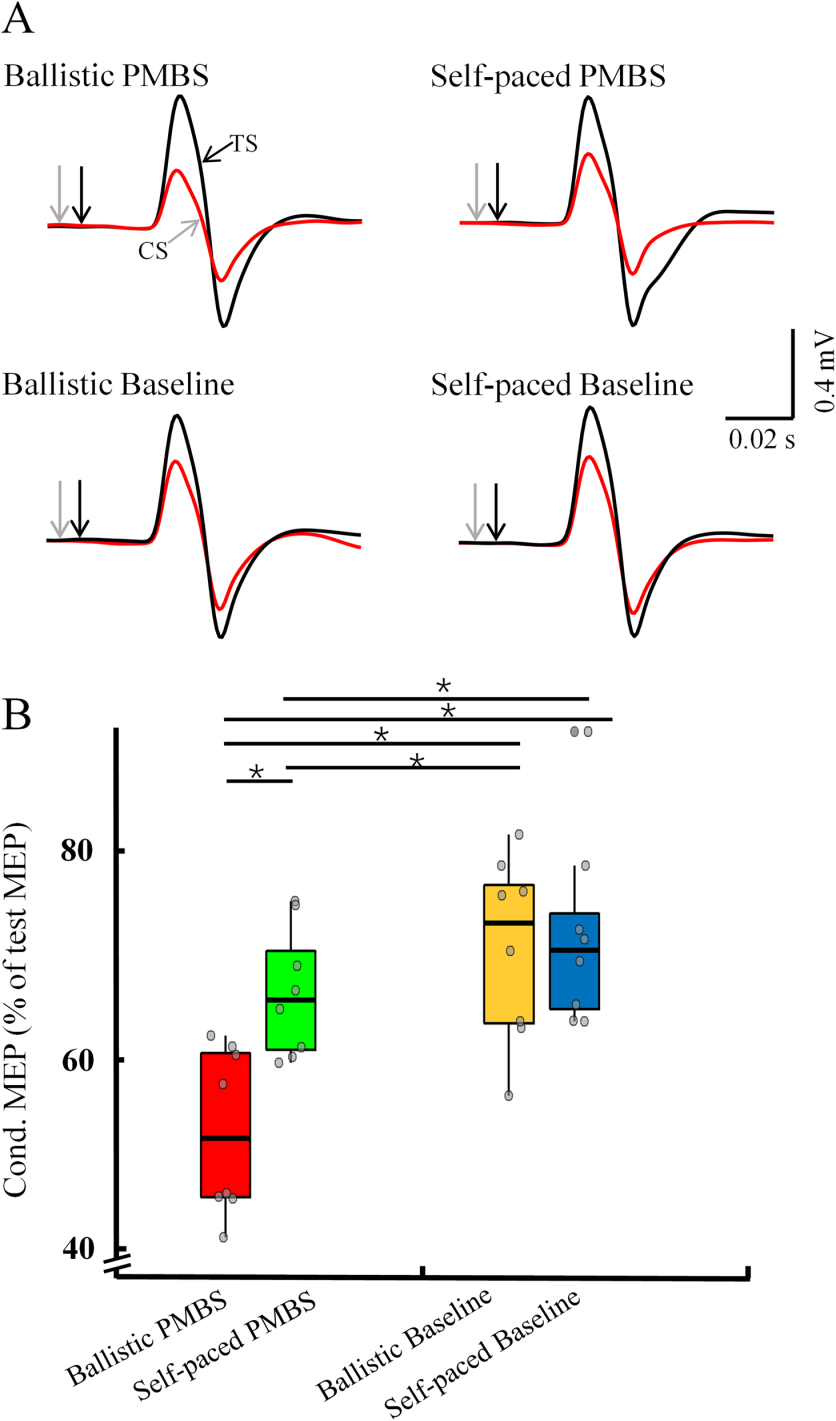
IHI across different conditions. ***A***, IHI from a representative subject (averaged across trials) during various conditions. ***B***, Group data. The abscissa shows different conditions, while the ordinate displays IHI across subjects, with each gray circle representing an individual value. Error bars indicate SEs. *p < 0.05; PMBS, postmovement beta synchronization; MEP, motor-evoked potential.

As illustrated in Figure 9, the analysis revealed that there were significant effects of speed (ballistic, self-paced; *F*_(1,31)_ = 16.406; *p* = 0.005), timing of TMS triggering (PMBS peak time, PMBS recovery baseline time; *F*_(1,31)_ = 73.142; *p* < 0.001), and their interaction (*F*_(1,31)_ = 16.415; *p* = 0.005) on IHI. Post hoc analysis showed that IHI during PMBS peak time was significantly strengthened compared with recovery baseline time under both ballistic movement (PMBS peak time, 0.52 ± 0.09; PMBS recovery baseline time, 0.72 ± 0.10; *p* < 0.001) and self-paced movement (PMBS peak time, 0.67 ± 0.07; PMBS recovery baseline time, 0.74 ± 0.11; *p* = 0.015). Moreover, IHI during ballistic movement was significantly more pronounced than during self-paced movement at PMBS peak time (*p* < 0.001). However, there was no significant difference in IHI between ballistic and self-paced movement during PMBS recovery time (*p* = 0.589). Therefore, we can infer that IM1 transmits more effective inhibitory regulation to CM1 during PMBS, which is enhanced under ballistic conditions compared with self-paced conditions. The results of ANOVAs of IHI are presented in Table 1.

**Figure 10.**
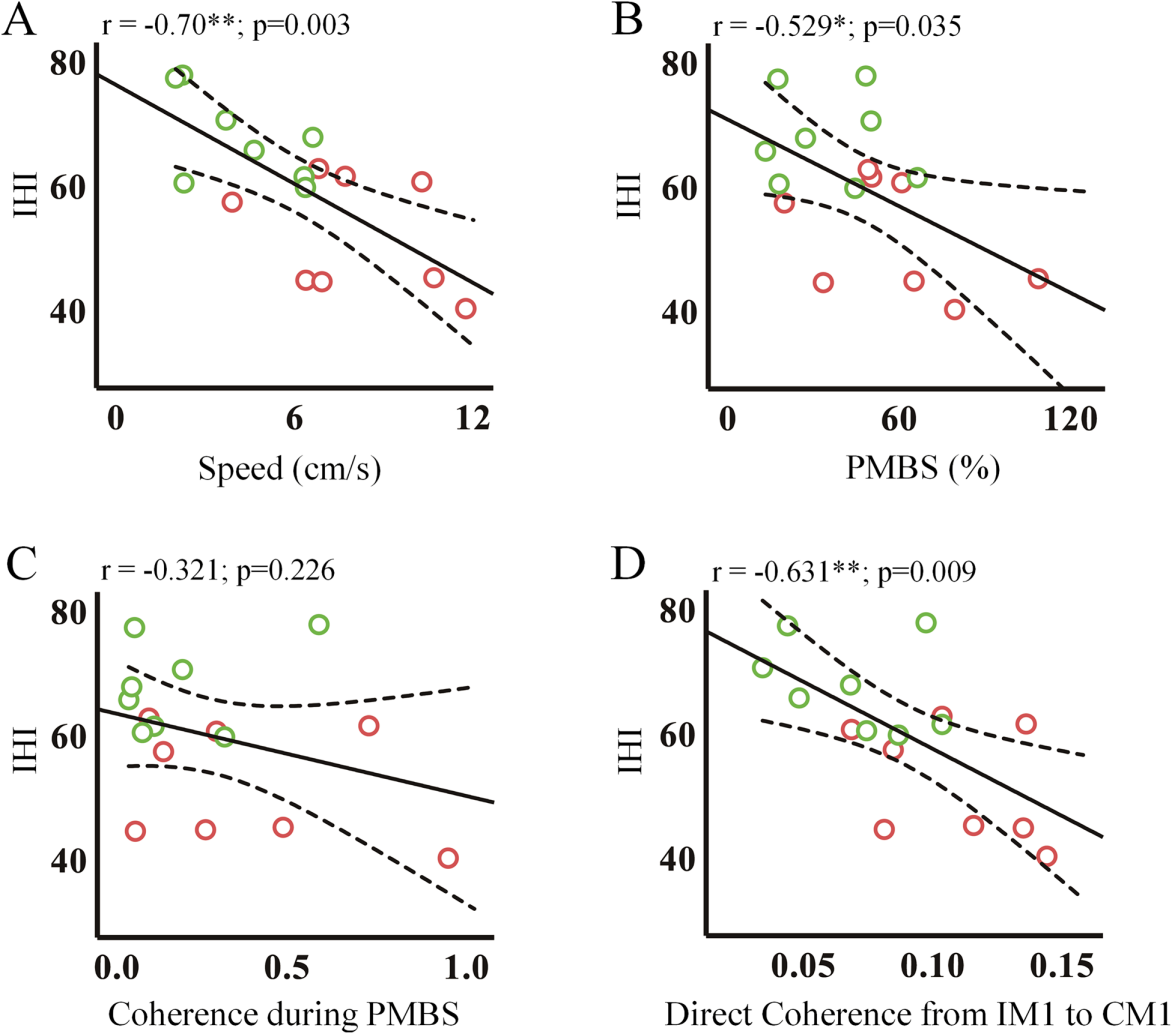
Scatterplots of significant associations. The relationships of IHI with speed (***A***), PMBS (***B***), coherence during PMBS (***C***), and directed coherence from IM1 to CM1 (***D***). The solid and dotted lines represent the regression lines and the 95% confidence limits, respectively. PMBS, postmovement beta synchronization; IM1, ipsilateral motor cortex; CM1, contralateral motor cortex; IHI, interhemispheric inhibition.

To explore the association of IHI with four parameters (speed, PMBS, coherence during PMBS, and directed coherence in the PMBS period), we computed the correlation coefficients using Pearson's correlation analysis (Fig. 10). A significant negative correlation was found between speed and IHI values (*r* = −0.70; *p* = 0.003), PMBS and IHI values (*r* = −0.529; *p* = 0.035), and directed coherence from IM1 to CM1 and IHI values (*r* = −0.631; *p* = 0.009). Larger PMBS values corresponded to smaller IHI values, indicating a stronger inhibitory effect from the right hemisphere to the left hemisphere. The results of the correlation between IHI during the MRBD and PMBS periods are presented in Tables 2 and 3, respectively.

## Discussion

### Interhemispheric asymmetry at different speeds

The present study demonstrated that MRBD is bilaterally present during movement execution, followed by bilateral PMBS, consistent with previous research (Little et al., 2019; Pfurtscheller and Da Silva, 1999; Wessel, 2020; Zhang et al., 2020, 2024). These findings corroborate that both MRBD and PMBS exhibit contralateral dominance during unilateral movement (Pfurtscheller et al., 1997; Leocani et al., 2001; Szurhaj et al., 2003; Rothwell et al., 2021; Wischnewski et al., 2022; Long et al., 2024), highlighting the role of beta oscillations in movement initiation and cancellation (Little et al., 2019; Wessel, 2020). Additionally, Correia et al. (2022) emphasized the importance of investigating fast limb movements. Our findings extend this understanding by demonstrating that PMBS, but not MRBD, is highly velocity-dependent. Larger PMBS was observed in ballistic movements compared with self-paced movements, consistent with previous studies (Zhang et al., 2020; Correia et al., 2022).

### Interhemispheric connection during the MRBD period

During the MRBD period, interhemispheric connections were evident during movement execution at different speeds. However, in the present study, the influence of speed was not observed. Coherence appears to be linked to motion complexity rather than specific motion tasks. Nevertheless, the impact of velocity on coherence remains a topic of debate. Toma et al. (2002) suggested that motor cortical activation and coupling were more pronounced during faster movements. However, Serrien and Brown (2002) found that the coupling between the primary sensorimotor cortices in the beta frequency band decreased with increasing movement speed. This effect was particularly strong in the antiphase mode compared with the in-phase mode. In the present study, the impact of speed on coherence was not detected, possibly due to differences in experimental design. The directed coherence analysis revealed that the predominant drive was from CM1 to IM1, consistent with previous studies (Amunts et al., 1996; Gross et al., 2005; Chettouf et al., 2020; Säisänen et al., 2021; Takasawa et al., 2022). This finding suggests that both hemispheres are involved in the execution of unilateral movements (Amunts et al., 1996; Gross et al., 2005; Beaulé et al., 2012; Chettouf et al., 2020; Merrick et al., 2022). Notably, interhemispheric coherence and spectral power desynchronization are distinct phenomena (Manganotti et al., 1998; Bundy et al., 2018), which may explain the lack of correlation observed between MRBD and coherence during movement execution.

### Interhemispheric connection during the PMBS period

The lack of postmotion bilateral coupling has been noted in previous studies (Leocani et al., 1997; Andrew and Pfurtscheller, 1999; Chettouf et al., 2020). Paradoxically, we observed a transient increase in interhemispheric coupling after motor termination. PMBS is task-dependent and closely related to motion parameters (Barone and Rossiter, 2021; Fry et al., 2016; Kang et al., 2023; Zhang et al., 2020, 2024). Therefore, the possible reason for the contradiction might be the variation within the task design.

An increase in task complexity is associated with enhanced intercerebral coherence (Gross et al., 2005; Aliakbaryhosseinabadi et al., 2021), which may explain the observed discrepancies. In contrast to studies investigating simple, self-paced movements (Leocani et al., 1997; Andrew and Pfurtscheller, 1999), we introduced more constrained and precisely timed tasks that demand higher motor control and coordination. These more challenging tasks may prompt the brain to exceed the threshold for interhemispheric interaction compared with simpler tasks. Another critical factor in motor cortical dynamics is movement speed. Previous studies primarily focused on self-paced movements without controlling for speed (Leocani et al., 1997; Andrew and Pfurtscheller, 1999). In contrast, our study tested two distinct conditions: ballistic (∼130 ms) and self-paced (∼180 ms). This highlights the pivotal role of speed in interhemispheric interactions, with earlier studies potentially lacking coherence due to their exclusive focus on self-paced movements (Leocani et al., 1997; Andrew and Pfurtscheller, 1999; Gross et al., 2005).

Our findings revealed a correlation between movement velocity and interhemispheric connectivity. During the PMBS period, directed coherence from IM1 to CM1 is stronger in ballistic movements than in self-paced movements, indicating greater functional coordination between IM1 and CM1. The increase in PMBS in faster movements may necessitate stronger interhemispheric connectivity. Prior studies have demonstrated that contralateral PMBS reflects cortical inhibition (Gilbertson et al., 2005; Pogosyan et al., 2009; Little et al., 2019; Hervault et al., 2021; Marques et al., 2022). However, coherence during PMBS only indicates that two regions are communicating, regardless of whether this communication is inhibitory or excitatory (Manganotti et al., 1998; Bundy et al., 2018). It is possible that interhemispheric connectivity during PMBS does not directly reflect the neuroregulatory mechanisms (excitability or inhibition) between IM1 and CM1. To further investigate this phenomenon, we employed paired-pulse TMS to assess IHI across the corpus callosum. The results, as illustrated in Figure 10, demonstrate a negative correlation between IHI and PMBS, coherence during PMBS, and directed coherence from IM1 to CM1. This indicates that interhemispheric connectivity reflects an inhibitory mechanism from the right to the left hemisphere during PMBS, with the ipsilateral hemisphere playing an active role in motor control. Furthermore, a negative correlation was observed between movement speed and IHI, indicating that faster movements are associated with stronger inhibition from IM1 to CM1 (Fig. 10*A*). Greater inhibition at higher speeds may assist in suppressing undesired mirror movements and enhancing the precision of motor commands (Ferbert et al., 1992; Meyer et al., 1995; Correia et al., 2022). Our findings lend support to the use of PMBS as a biomarker for movement control and suggest the potential for the development of brain stimulation technologies to enhance motor-related neural circuits, thereby promoting rapid recovery through increased neural activity and plasticity.

The greater directed coherence from IM1 to CM1 in ballistic movements compared with self-paced movements during the PMBS period suggests a significant role for the ipsilateral hemisphere in movement control. This coherence may reflect an inhibitory mechanism from the right to the left hemisphere. Reinforced suppression of IM1 to CM1 in ballistic movements potentially reduces unwanted or residual right-hand movements (Hübers et al., 2008; Cabibel et al., 2020; Iwama et al., 2020). This would support motor stability of the motor system, which aligns with our previous findings on PMBS (Zhang et al., 2020, 2024).

### Practical utility

The present study elucidates the excitatory and inhibitory neurophysiological mechanisms of PMBS and the role in across hemispheric information exchange. Together with previous studies from our group, the robustness of PMBS as a biomarker for the stabilization of the movement system has been repeatedly demonstrated (Zhang et al., 2020, 2024). Therefore, PMBS may have the potential to serve as a reliable neural index for brain–computer interface (BCI) paradigms. Through a real-time update module, we can monitor the temporal dynamics of PMBS and decode the state of the movement system. This constitutes the core module of a closed-loop, brain-controlled repetitive TMS (rTMS) system with the potential to enhance postapoplectic motor rehabilitation. Furthermore, using source reconstruction algorithms such as weighted minimum-norm estimation, PMBS can be localized to cortical and subcortical regions, offering a neurophysiological foundation for the optimization of target areas in rTMS and deep brain stimulation interventions (Molins et al., 2008; Chang et al., 2015; Krishnaswamy et al., 2017; Li et al., 2019).

## Conclusion

The present study demonstrated that speed not only exerts control over PMBS but also influences interhemispheric functional connectivity, thereby regulating IHI during the PMBS period. The occurrence of ballistic movements has been observed to result in increased suppression from IM1 to CM1, thereby underscoring the pivotal role of the ipsilateral hemisphere in motor control. The findings in the present study highlight the potential of PMBS as a biomarker of motor state, which may pave the way for the development of a PMBS-driven closed–loop rTMS system for postapoplectic motor rehabilitation via BCI paradigms.

## Data Availability

The raw and preprocessed data generated in this study have been deposited in a local database. The EEG and EMG data are available under restricted access as they contain personally identifiable information, and patients have not consented to data distribution. Access can be obtained from the corresponding author upon reasonable request. The code for this article can be accessed online at https://github.com/Morinooku/code-for-eN-TNWR-0370-24X-.git.

## Synthesis

Reviewing Editor: Mark Laubach, American University Decisions are customarily a result of the Reviewing Editor and the peer reviewers coming together and discussing their recommendations until a consensus is reached. When revisions are invited, a fact-based synthesis statement explaining their decision and outlining what is needed to prepare a revision will be listed below. The following reviewer(s) agreed to reveal their identity: NONE. Note: If this manuscript was transferred from JNeurosci and a decision was made to accept the manuscript without peer review, a brief statement to this effect will instead be what is listed below.

Two experts reviewed your manuscript and found it be an interesting study that is worthy of publication provided that extensive revisions are made.

Reviewer 1 wrote: "This is an area that has been covered before, the authors cite a good part of the existing literature and provide some modest advances on interhemispheric coupling in PMBS."

Reviewer 2 wrote: "This manuscript advances our understanding of interhemispheric connectivity and the role of PMBS during motor tasks, with emphasis on the influence of movement speed. However, more clarification is needed regarding the underlying neuromodulation mechanisms."

Full reviews are given below. In addition, essential revisions that following a discussion of your paper among the reviewers are included below. Please revise to address all point that were raised.

Reviewer 1

Zhang and colleagues present data on interhemispheric coherence in beta frequencies during finger movements and compare ballistic and self-paced conditions. They show increased interhemispheric coherence during PMBS, along with a movement speed effect. They also study interhemispheric inhibition using a paired TMS pulse design and showed enhancement of this inhibition IM1 to CM1 during the PMBS, also with a movement speed effect.

This is an area that has been covered before, the authors cite a good part of the existing literature and provide some advances. The study and data appear solid. I have two main comments and then a few small suggestions.

1. In general, the study has a relatively small subject sample and appears to use relatively few trials compared to what I might expect in the field. For example, 8 subjects feels low. To justify this, I think the authors should include some calculation of whether they have the statistical power to answer the questions they are posing.

2. During the MRBD, in many studies one can see that there can be no detectable beta power at all in the spectrum during this phase. In such cases, measuring coherence in the beta spectrum makes no sense - there is nothing to cohere. So to validate analyses of coherence and dir coh between regions during this period, the control analysis to show that there remains sufficient power above zero is required.

Other comments

The abstract does not mention the types of recordings or indeed subjects, and this should be added.

There is some variability of terminology throughout the manuscript that could be usefully cleaned up - "an increase in ERSP" is really an increase in the spectral power, and ERS and PMBS seem to be used interchangeably.

A small further control analysis should be included. All effects are measured relative to a pre-trial baseline. An important confirmation is to show that there are no effects of beta power or coherence *within* this baseline, to ensure that the measures are specific to the post movement timepoints discussed. This is particularly true as this is a block design, so trials of the movement conditions are not interleaved.

I haven't checked the data policy of the journal, but the data availability statement ("reasonable request") seems disappointing. It is perfectly possible to anonymise these data and make them available after publication, I would encourage the authors to do so.

Typos etc

P26 Missing letter in section title "Correlation"

Description of Fig 6 P27, DEF refer to PMBS but the text mentions MRBD

P32 First title "Speeds"

Reviewer 2

1. Movement Speed and Interhemispheric Coupling:

• The results indicating stronger coherence during ballistic movements in the PMBS phase are compelling. However, the discrepancy with previous studies (Gross et al., 2005) that report a lack of bilateral coupling in this phase should be addressed more thoroughly. Could task-specific factors such as the complexity of the movement or task difficulty account for this variation? Additional comparisons with earlier work will strengthen the manuscript's interpretation.

2. Inhibition and Speed Correlation:

• The negative correlation between speed and IHI during the PMBS phase is an exciting finding. However, the manuscript would benefit from a deeper discussion of the functional implications of stronger IHI from IM1 to CM1 at faster speeds. How might this influence motor control or learning in clinical populations?

3. Causality Direction (IM1 to CM1):

• The observed increase in directed coherence from IM1 to CM1 during ballistic movement raises intriguing questions about the functional role of the ipsilateral hemisphere. The manuscript should explore possible hypotheses as to why IM1 takes on a dominant role in regulating movement post-termination.

4. Beta Oscillations and Task Design:

• The inclusion of beta oscillations during both the MRBD and PMBS periods contributes to the novelty of the study. However, the discussion on beta activity should integrate more recent findings on beta bursts (e.g., Wessel, 2020) and their role in movement inhibition.

5. TMS Methodology:

• The TMS protocol used to assess IHI is appropriate, but further elaboration on how stimulation intensities were chosen and their potential variability across subjects would help clarify the robustness of the results. Were intensities adjusted based on individual motor thresholds or were standard protocols used?

6. Cohesion between Methods and Results:

• While the methods are well-described, the results section feels slightly dense. Consider streamlining some of the descriptions of statistical tests to enhance readability. It might be helpful to include summary tables for the most important statistical outcomes, making it easier for readers to quickly grasp key findings.

7. Potential Applications:

• The practical applications of these findings in motor rehabilitation or brain-computer interfaces should be highlighted more clearly. Could enhancing interhemispheric coupling through targeted stimulation improve motor recovery post-stroke?

Statistics: The statistical analysis appears to be methodologically sound. The use of repeated-measures ANOVA and correlation analyses is appropriate, but the manuscript should provide more justification for the use of a linear model to describe the relationship between speed and neural metrics. It might be useful to explore non-linear models, as the relationships could be more complex.

Extended Data: Extended data sets, such as coherence and directed coherence analysis, are critical to this study. These should be included as they support the key results, especially regarding the differences in coherence across the MRBD and PMBS periods. However, a brief explanation of how coherence values were processed across subjects would enhance clarity.

Software: The manuscript mentions the use of EEGLab for ICA and Spike2 for EMG analysis. While these are standard, a brief description of any custom code or pre-processing scripts used would be helpful. If possible, sharing the code would further support reproducibility.

In addition, discussion and consensus between the reviewers led to these additional points that you should address:

1. Sample Size and Statistical Power

To ensure the credibility of the results, the authors should provide a statistical power calculation to demonstrate whether the current sample size is adequate to answer the research questions posed. This would help alleviate concerns about potential bias due to the limited number of subjects.

2. Beta Power During the MRBD Phase

The authors should conduct a control analysis to ensure there is sufficient power above zero during the MRBD phase, thus validating the coherence and directed coherence analyses between regions during this period.

3. Baseline Period Analysis

A baseline period control analysis should be included. This would confirm that no significant effects of beta power or coherence are present within the baseline period, ensuring that the measured effects are specific to the post-movement time points. Given the block design of this study, where trials are not interleaved, this is especially crucial for the robustness of the findings.

4. Terminology Consistency

There were inconsistencies in terminology throughout the manuscript. Terms such as "an increase in ERSP" should be clarified as "an increase in spectral power," and ERS and PMBS should not be used interchangeably. Cleaning up the terminology will greatly improve the manuscript's clarity and readability.

5. Data Availability

Regarding the data availability statement, and given that the data can be anonymized, the authors should make the data publicly available, which aligns with open science principles and could enhance the reproducibility of their work.

6. TMS Methodology and Stimulation Intensity

The authors should provide more details on how the stimulation intensities were chosen and discuss the potential variability across subjects. Additional details in this area would help clarify the robustness of the results.

7. Relationship Between PMBS and Movement Speed

The results showing stronger coherence during ballistic movements in the PMBS phase are intriguing. The authors should delve deeper into the reasons for this discrepancy, potentially exploring factors like task complexity or difficulty.

## Author Response

Dear Editor, On behalf of my co-authors, I would like to extend our sincere appreciation to you and the reviewers for the thorough and insightful feedback provided on our manuscript (Manuscript ID: eN-TNWR-0370-24X). We have carefully considered each of the reviewers' suggestions and have made significant revisions to enhance the quality of the manuscript. In particular, we have thoroughly revised the text to improve the clarity of the experimental procedures and ensure that the interpretation of the results is as precise and accurate as possible. We believe these revisions have substantially strengthened the manuscript. Below, we have provided a detailed, point-by-point response to each of the reviewers' comments (in bold italics), and all revisions in the manuscript have been highlighted in red for ease of review.

My co-authors and I would like to express our sincere appreciation for the time and effort you and the reviewers have invested in evaluating our revised manuscript. We are hopeful that the revisions we have made effectively address all concerns raised, and we respectfully submit the manuscript for further consideration. We look forward to receiving your feedback and remain optimistic that the manuscript will now meet the standards for publication. Thank you once again for your invaluable guidance and support throughout this process.

Sincerely, Jinyi Long Reviewer #1 (Comments to the Author):

Zhang and colleagues present data on interhemispheric coherence in beta frequencies during finger movements and compare ballistic and self-paced conditions. They show increased interhemispheric coherence during PMBS, along with a movement speed effect. They also study interhemispheric inhibition using a paired TMS pulse design and showed enhancement of this inhibition IM1 to CM1 during the PMBS, also with a movement speed effect.

This is an area that has been covered before, the authors cite a good part of the existing literature and provide some advances. The study and data appear solid. I have two main comments and then a few small suggestions.

In general, the study has a relatively small subject sample and appears to use relatively few trials compared to what I might expect in the field. For example, 8 subjects feels low. To justify this, I think the authors should include some calculation of whether they have the statistical power to answer the questions they are posing.

Response:

We would like to express our sincerest gratitude to the reviewer for their invaluable feedback. In order to enhance the credibility and robustness of the article, we have incorporated the following additional explanations in accordance with the principles of scientific rigour. "The present study is a preliminary, small-sample investigation. The sample size was determined based on previous studies employing similar neurophysiological techniques (Stancák and Pfurtscheller, 1996; Leocani et al., 1997; Manganotti et al., 1998; Mima et al., 2000; Serrien and Brown, 2002; Toma et al., 2002; Szurhaj et al., 2003; Gross et al., 2005; Yao et al., 2007; Stevenson et al., 2011; Qiu et al., 2016; Little et al., 2019). The mean number of participants in these studies was approximately 7.33. We initially recruited 10 volunteers; however, two withdrew due to personal circumstances. Ultimately, eight subjects completed the experiment, which exceeds the average sample size of the aforementioned studies" (Page 8-9) "The configuration of the experimental procedure was adapted from previous studies (Ferbert et al., 1992; Yao et al., 2007; Stevenson et al., 2011; Fry et al., 2016; Qiu et al., 2016) and is presented in Fig. 1A" (Page 10-11) "The number of trials employed in the present study is consistent with those used in previous research (Ferbert et al., 1992; Fry et al., 2016). Furthermore, we calculated the time-frequency representation, which has been demonstrated to exhibit a superior signal-to-noise ratio compared to event-related potentials (Baker et al., 1997; Mohammadi et al., 2020; Arts and van den Broek, 2022; Morales and Bowers, 2022)" (Page 11) ----------------------------------------------------------------------------------------------------------------- During the MRBD, in many studies one can see that there can be no detectable beta power at all in the spectrum during this phase. In such cases, measuring coherence in the beta spectrum makes no sense - there is nothing to cohere. So to validate analyses of coherence and dir coh between regions during this period, the control analysis to show that there remains sufficient power above zero is required.

Response:

We sincerely appreciate the reviewer's insightful comment regarding the potential limitations of measuring coherence during the MRBD phase.

We defined MRBD in the introduction part: "Beta ERSP encompasses a decreased beta power change relative to the baseline, namely movement related beta desynchronization (MRBD), followed by an increase in beta power after the termination of movement, known as post-movement beta synchronization (PMBS) (Deiber et al., 2015; Pfurtscheller and Da Silva, 1999; Qiu et al., 2016)" (Page 5) MRBD is not indicated by the absolute value of beta power but by the ratio of beta power during the MRBD period to the average baseline power. However, the sentence in the "Time-Frequency Representation (TFR)" section, "EEG power at each frequency and time point was normalized by dividing it with the average power during baseline" may be somewhat ambiguous. To improve clarity and ensure comprehension, we have revised this section for enhanced readability. "The EEG power at each frequency and time point was normalized by subtracting the average power during the baseline period and then dividing it by the average baseline power. The resulting value was then multiplied by 100, thus expressing the event-related EEG power change relative to the baseline. The aforementioned procedure is described in detail by Nakayashiki et al. (2014).:

P_rest represents the mean power spectrum during the rest period (T_rest) and relative power at each time point and the relative power at each time point (RP(n)) was calculated using the instantaneous power spectrum at each time point (P_n)" (Page 16) ----------------------------------------------------------------------------------------------------------------- The abstract does not mention the types of recordings or indeed subjects, and this should be added.

Response:

The reviewer's astute observation regarding the abstract has been duly noted and addressed in the revised version, which now includes details about the types of recordings and subjects. "In the present study, we investigated eight healthy, right-handed volunteers using a combination of electroencephalography (EEG), transcranial magnetic stimulation (TMS), and electromyography (EMG)" (Page 3) ----------------------------------------------------------------------------------------------------------------- There is some variability of terminology throughout the manuscript that could be usefully cleaned up "an increase in ERSP" is really an increase in the spectral power, and ERS and PMBS seem to be used interchangeably.

Response:

We sincerely appreciate the reviewer's careful attention to the terminology in our manuscript. To improve clarity, we have replaced "ERD" and "ERS" with MRBD and PMBS to specifically indicate beta power activity. ERSP is mentioned only in the introduction to set the stage for MRBD and PMBS, minimizing any potential confusion.

Changing "Event-related spectral perturbation (ERSP), including bilateral movement-related beta desynchronization (MRBD) and post-movement beta synchronization (PMBS)" to "Beta event-related spectral perturbation (ERSP), including bilateral movement-related beta desynchronization (MRBD) and post-movement beta synchronization (PMBS)" (Page 3) Changing " Reflected by electroencephalography (EEG) signals, this phenomenon is represented by an event-related spectral perturbation (ERSP) " to "Reflected by electroencephalography (EEG) signals, this phenomenon is represented by an beta event-related spectral perturbation (ERSP)" (Page 5) Changing "ERSP encompasses a decreased power change relative to the baseline, namely movement related beta desynchronization (MRBD), followed by an increase in ERSP after the termination of movement, known as post-movement beta synchronization (PMBS)" to "Beta ERSP encompasses a decreased beta power change relative to the baseline, namely movement related beta desynchronization (MRBD), followed by an increase in beta power after the termination of movement, known as post-movement beta synchronization (PMBS)" (Page 5) Changing "The association between coherence and ERSP over different time courses has been investigated in previous studies" to "The association between coherence and MRBD and PMBS over different time courses has been investigated in previous studies" (Page 5) Changing "Leocani et al. reported an increase in coherence over sensorimotor and frontal areas during the ERD phase" to "Leocani et al. (1997) reported an increase in coherence over sensorimotor and frontal areas during the MRBD phase" (Page 5) Changing "However, increasing evidence suggests that the motor cortex does not remain in an idle state during the beta ERS period but is involved in motor modulation" to "However, increasing evidence suggests that the motor cortex does not remain in an idle state during the PMBS period but is involved in motor modulation" (Page 6) Changing "An increase in beta rhythm power (i.e., PMBS) could be observed after the isometric abduction movement" to "PMBS could be observed after the isometric abduction movement" (Page 11) Changing "The ERSP waveforms, which include two stages: MRBD during the execution of the motion and PMBS after movement while performing the task" to "The beta waveforms, which include two stages: MRBD during the execution of the motion and PMBS after movement while performing the task" (Page 12) Changing "The latency of beta rebound was identified as the time at which beta power increased by more than 10% above baseline" to "The latency of PMBS was identified as the time at which beta power increased by more than 10% above baseline" (Page 17) Changing "Repeated measures two-way ANOVAs were also conducted to study the effect of speed (ballistic, self-paced) and time course of ERSP (MRBD period, PMBS period) on interhemispheric coherence and interhemispheric directed coherence" to "Repeated measures two-way ANOVAs were also conducted to study the effect of speed (ballistic, self-paced) and time course (MRBD period, PMBS period) on interhemispheric coherence and interhemispheric directed coherence" (Page 21) Changing "Repeated-measures ANOVA showed a significant effect of speed (ballistic, self-paced) (F(1,31) = 12.030, p = 0.005, ANOVA) and time course of ERSP (MRBD period, PMBS period) (F(1,31) = 14.485, p = 0.003, ANOVA) with a significant effect of their interaction(F(1,31) = 7.788, p = 0.016, ANOVA)" to "As illustrated in Fig. 4, the coherence between CM1 and IM1 was quantified. There was no significant difference in coherence between ballistic and self-paced movements (F(1,14) = 0.149, p = 0.705). Therefore, baseline analyses were not further pursued. Significant effects of speed (ballistic, self-paced) (F(1,31) = 12.030, p = 0.005) and time course (MRBD period, PMBS period) (F(1,31) = 14.485, p = 0.003) were observed, as well as a significant effect of their interaction (F(1,31) = 7.788, p = 0.016) (Fig. 4C)" (Page 25-26) Changing "Directed coherence at different time points of ERSP" to "Directed coherence at different time points of MRBD and PMBS" (Page 26) Chnaging "Fig. 5 presents the results of directed coherence during different time courses of ERSP" to "Fig. 5 presents the results of directed coherence during different time courses of MRND and PMBS" (Page 27) ----------------------------------------------------------------------------------------------------------------- A small further control analysis should be included. All effects are measured relative to a pre-trial baseline. An important confirmation is to show that there are no effects of beta power or coherence *within* this baseline, to ensure that the measures are specific to the post movement timepoints discussed. This is particularly true as this is a block design, so trials of the movement conditions are not interleaved.

Response:

We sincerely thank the reviewer for their thoughtful observation regarding the potential influence of baseline coherence on our post-movement findings. Coherence between C3 and C4 during the preparatory period is indeed a well-documented phenomenon in the literature (Pfurtscheller and Andrew, 1999; Fronso et al., 2018). In response to this important point, we conducted additional analyses which revealed no significant differences between-group of different speeds in preparatory coherence levels: "There was no significant difference in coherence between ballistic and self-paced movements (F(1,14) = 0.149, p = 0.705). Therefore, baseline analyses were not further pursued" (Page 25) ----------------------------------------------------------------------------------------------------------------- I haven't checked the data policy of the journal, but the data availability statement ("reasonable request") seems disappointing. It is perfectly possible to anonymise these data and make them available after publication, I would encourage the authors to do so.

Response:

I am grateful for your perceptive observation regarding our data availability statement. We are grateful for your encouragement to make our data more openly accessible and recognize the vital role that data sharing plays in advancing scientific research and enhancing reproducibility. Our decision to make data available "upon reasonable request" is based on a number of important considerations that we believe are essential to address.

It is of the utmost importance that we safeguard the privacy of our study participants. The EEG and EMG data contain sensitive, personally identifiable information, and our participants have not explicitly consented to the broad distribution of their data. Although we are aware of the existence of anonymization techniques, we recognize that ensuring complete de-identification of complex neurophysiological data while maintaining its scientific integrity presents a significant challenge.

Furthermore, it should be noted that this study is part of an ongoing series of investigations. The data collected here are integral to further analyses currently in progress, including the application of machine learning techniques. We are eager to ascertain the insights that these supplementary analyses may provide, and we are assiduously preparing a manuscript that will delineate these findings. In light of the aforementioned considerations, we hope you can appreciate our caution regarding premature data release, as it could potentially impact the novelty of our forthcoming work.

We would like to reiterate our unwavering commitment to the principles of open science and data sharing. It is not our intention to withhold data indefinitely. Once our ongoing analyses are complete and the related manuscripts have been published, we will engage in discussions with our participants and obtain consent for the broad distribution of the anonymized data. Subsequently, we anticipate making the complete dataset accessible via suitable scientific data repositories. We believe that this approach allows us to reconcile our ethical obligations to research participants, maintain the integrity of our ongoing work, and ultimately make a more comprehensive contribution to the scientific community.

----------------------------------------------------------------------------------------------------------------- Typos etc (P26 Missing letter in section title "Correlation"; Description of Fig 6 P27, DEF refer to PMBS but the text mentions MRBD; P32 First title "Speeds") Response:

We are grateful to the reviewer for their meticulous attention to detail. We extend our sincerest apologies for these inadvertent oversights and are grateful for the opportunity to rectify them.

We have corrected the section title to read "Correlation Analysis during MRBD and PMBS Period" (Page 28) We changed the text "Fig. 6DEF revealed positive correlations between MRBD and speed" to the correct text "Fig. 6DEF revealed positive correlations between PMBS and speed" (Page 28) We have corrected the section title to read "Interhemispheric Asymmetry at Different Speeds" (Page 34) Reviewer #2 (Comments to the Author):

Movement Speed and Interhemispheric Coupling:

The results indicating stronger coherence during ballistic movements in the PMBS phase are compelling. However, the discrepancy with previous studies (Gross et al., 2005) that report a lack of bilateral coupling in this phase should be addressed more thoroughly. Could task-specific factors such as the complexity of the movement or task difficulty account for this variation? Additional comparisons with earlier work will strengthen the manuscript's interpretation.

Response:

We are grateful for your insightful feedback on our manuscript. In response to the observation regarding the discrepancy with previous studies, particularly that of Gross et al. (2005), we would like to address this concern.

Upon re-examining Gross et al. (2005), we found that their study does not explicitly report a lack of bilateral coupling during the PMBS phase. Their presentation, particularly Figure 4(C), lacks specificity regarding which task conditions (rest, hold l, hold r, wrist l, wrist r, p, ap) are represented, making direct comparisons difficult. We sincerely appreciate the reviewer's insight regarding task-specific factors like movement complexity and difficulty. In response, we have expanded our discussion and made the following revisions. "An increase in task complexity is associated with enhanced inter-cerebral coherence (Gross et al., 2005; Aliakbaryhosseinabadi et al., 2021), which may explain the observed discrepancies. In contrast to studies investigating simple, self-paced movements (Andrew and Pfurtscheller, 1999; Leocani et al., 1997), we introduced more constrained and precisely timed tasks that demand higher motor control and coordination. These more challenging tasks may prompt the brain to exceed the threshold for interhemispheric interaction compared to simpler tasks. Another critical factor in motor cortical dynamics is movement speed. Previous studies primarily focused on self-paced movements without controlling for speed (Andrew and Pfurtscheller, 1999; Leocani et al., 1997). In contrast, our study tested two distinct conditions: ballistic (~130 ms) and self-paced (~180 ms). This highlights the pivotal role of speed in interhemispheric interactions, with earlier studies potentially lacking coherence due to their exclusive focus on self-paced movements (Gross et al., 2005; Andrew and Pfurtscheller, 1999; Leocani et al., 1997)" (Page 37) ----------------------------------------------------------------------------------------------------------------- Inhibition and Speed Correlation:

The negative correlation between speed and IHI during the PMBS phase is an exciting finding. However, the manuscript would benefit from a deeper discussion of the functional implications of stronger IHI from IM1 to CM1 at faster speeds. How might this influence motor control or learning in clinical populations? Response:

We sincerely appreciate the opportunity to address this important point. In response to your valuable suggestion, we have revised our manuscript to provide a more comprehensive analysis, particularly highlighting its potential impact on motor control in clinical populations. "Our findings revealed a correlation between movement velocity and interhemispheric connectivity. During the PMBS period, directed coherence from IM1 to CM1 is stronger in ballistic movements than in self-paced movements, indicating greater functional coordination between IM1 and CM1. The increase in PMBS in faster movements may necessitate stronger interhemispheric connectivity. Prior studies have demonstrated that contralateral PMBS reflects cortical inhibition (Gilbertson et al., 2005; Pogosyan et al., 2009; Little et al., 2019; Hervault et al., 2021; Marques et al., 2022). However, coherence during PMBS only indicates that two regions are communicating, regardless of whether this communication is inhibitory or excitatory (Manganotti et al., 1998; Bundy et al., 2018). It is possible that interhemispheric connectivity during PMBS does not directly reflect the neuroregulatory mechanisms (excitability or inhibition) between IM1 and CM1. To further investigate this phenomenon, we employed paired-pulse TMS to assess IHI across the corpus callosum. The results, as illustrated in Fig. 10, demonstrate a negative correlation between IHI and PMBS, coherence during PMBS, and directed coherence from IM1 to CM1. This indicates that interhemispheric connectivity reflects an inhibitory mechanism from the right to the left hemisphere during PMBS, with the ipsilateral hemisphere playing an active role in motor control. Furthermore, a negative correlation was observed between movement speed and IHI, indicating that faster movements are associated with stronger inhibition from IM1 to CM1 (Fig. 10A). Greater inhibition at higher speeds may assist in suppressing undesired mirror movements and enhancing the precision of motor commands (Ferbert et al., 1992; Meyer et al., 1995; Correia et al., 2022)" (Page 37-38) ----------------------------------------------------------------------------------------------------------------- Causality Direction (IM1 to CM1):

The observed increase in directed coherence from IM1 to CM1 during ballistic movement raises intriguing questions about the functional role of the ipsilateral hemisphere. The manuscript should explore possible hypotheses as to why IM1 takes on a dominant role in regulating movement post-termination.

Response:

We sincerely appreciate your valuable feedback regarding the need for a deeper exploration of the functional role of the ipsilateral hemisphere. In response, we have expanded our discussion section to clarify the reasons why IM1 assumes a dominant role in regulating movement post-termination. "The greater directed coherence from IM1 to CM1 in ballistic movements compared to self-paced movements during the PMBS period suggests a significant role for the ipsilateral hemisphere in movement control. This coherence may reflect an inhibitory mechanism from the right to the left hemisphere. Reinforced suppression of IM1 to CM1 in ballistic movements potentially reducing unwanted or residual right-hand movements (Hübers et al., 2008; Cabibel et al., 2020; Iwama et al., 2020). This would support motor stability of the motor system, which aligns with our previous findings on PMBS (Zhang et al., 2020; Zhang et al., 2024)" (Page 39) ----------------------------------------------------------------------------------------------------------------- Beta Oscillations and Task Design:

The inclusion of beta oscillations during both the MRBD and PMBS periods contributes to the novelty of the study. However, the discussion on beta activity should integrate more recent findings on beta bursts (e.g., Wessel, 2020) and their role in movement inhibition.

Response:

We sincerely appreciate the reviewer's insightful suggestion to integrate more recent findings on beta bursts and their role in movement inhibition. MRBD and PMBS can also be interpreted as manifestations of beta bursts (Keinrath et al., 2006; Feingold et al., 2015). In response, we have incorporated recent studies (including study of Wessel (2020)) that explore this topic and report similar findings, thereby enhancing the context of our research. The following sections detail where these newer publications have been included. "The present study demonstrated that MRBD is bilaterally present during movement execution, followed by bilateral PMBS, consistent with previous research (Pfurtscheller and Da Silva, 1999; Little et al., 2019; Wessel, 2020; Zhang et al., 2020; Zhang et al., 2024). These findings corroborate that both MRBD and PMBS exhibit contralateral dominance during unilateral movement (Pfurtscheller et al., 1997; Leocani et al., 2001; Szurhaj et al., 2003; Rothwell et al., 2021; Wischnewski et al., 2022; Long et al., 2024), highlighting the role of beta oscillations in movement initiation and cancellation (Little et al., 2019; Wessel, 2020). Additionally, Correia et al. (2022) emphasized the importance of investigating fast limb movements. Our findings extend this understanding by demonstrating that PMBS, but not MRBD, is highly velocity-dependent. Larger PMBS was observed in ballistic movements compared to self-paced movements, consistent with previous studies (Zhang et al., 2020; Correia et al., 2022)" (Page 34) "The directed-coherence analysis revealed that the predominant drive was from CM1 to IM1, consistent with previous studies (Amunts et al., 1996; Gross et al., 2005; Chettouf et al., 2020; Säisänen et al., 2021; Takasawa et al., 2022). This finding suggests that both hemispheres are involved in the execution of unilateral movements (Amunts et al., 1996; Gross et al., 2005; Beaulé et al., 2012; Chettouf et al., 2020; Merrick et al., 2022). Notably, interhemispheric coherence and spectral power desynchronization are distinct phenomena (Manganotti et al., 1998; Bundy et al., 2018), which may explain the lack of correlation observed between MRBD and coherence during movement execution" (Page 36) "The lack of post-motion bilateral coupling has been noted in previous studies (Andrew and Pfurtscheller, 1999; Leocani et al., 1997; Chettouf et al., 2020)" (Page 37) "PMBS is task-dependent and closely related to motion parameters (Fry et al., 2016; Zhang et al., 2020; Barone and Rossiter, 2021; Kang et al., 2023; Zhnag et al., 2024)" (Page 37) "An increase in task complexity is associated with enhanced inter-cerebral coherence (Gross et al., 2005; Aliakbaryhosseinabadi et al., 2021), which may explain the observed discrepancies" (Page 37) "This highlights the pivotal role of speed in interhemispheric interactions, with earlier studies potentially lacking coherence due to their exclusive focus on self-paced movements (Gross et al., 2005; Andrew and Pfurtscheller, 1999; Leocani et al., 1997)" (Page 37) "Prior studies have demonstrated that contralateral PMBS reflects cortical inhibition (Gilbertson et al., 2005; Pogosyan et al., 2009; Little et al., 2019; Hervault et al., 2021; Marques et al., 2022). However, coherence during PMBS only indicates that two regions are communicating, regardless of whether this communication is inhibitory or excitatory (Manganotti et al., 1998; Bundy et al., 2018)" (Page 38) "Greater inhibition at higher speeds may assist in suppressing undesired mirror movements and enhancing the precision of motor commands (Ferbert et al., 1992; Meyer et al., 1995; Correia et al., 2022)" (Page 38) "The greater directed coherence from IM1 to CM1 in ballistic movements compared to self-paced movements during the PMBS period suggests a significant role for the ipsilateral hemisphere in movement control. This coherence may reflect an inhibitory mechanism from the right to the left hemisphere. Reinforced suppression of IM1 to CM1 in ballistic movements potentially reducing unwanted or residual right-hand movements (Hübers et al., 2008; Cabibel et al., 2020; Iwama et al., 2020). This would support motor stability of the motor system, which aligns with our previous findings on PMBS (Zhang et al., 2020; Zhang et al., 2024)" (Page 39) "This coherence may reflect an inhibitory mechanism from the right to the left hemisphere, with reinforced suppression of IM1 to CM1 in ballistic movements potentially reducing unwanted or residual right-hand movements (Hübers et al., 2008; Cabibel et al., 2020; Iwama et al., 2020). This would support motor stability of the motor system, which aligns with our previous findings on PMBS (Zhang et al., 2020; Zhang et al., 2024)" (Page 39) The following are the newly included publications. "Aliakbaryhosseinabadi S, Lontis R, Farina D, Mrachacz-Kersting N (2021) Effect of motor learning with different complexities on EEG spectral distribution and performance improvement. Biomed Signal Process Control 66:102447.

Barone J, Rossiter HE (2021) Understanding the role of sensorimotor beta oscillations. Front Syst Neurosci 15:655886.

Bundy DT, Szrama N, Pahwa M, Leuthardt EC (2018) Unilateral, 3D arm movement kinematics are encoded in ipsilateral human cortex. J Neurosci 38:10042-10056.

Cabibel V, Hordacre B, Perrey S (2020) Implication of the ipsilateral motor network in unilateral voluntary muscle contraction: the cross-activation phenomenon. J Neurophysiol 123:2090-:2098.

Chettouf S, Rueda-Delgado LM, de Vries R, Ritter P, Daffertshofer A (2020) Are unimanual movements bilateral?. Neurosci Biobehav Rev 113:39-50.

Hervault M, Zanone PG, Buisson JC, Huys R (2021) Cortical sensorimotor activity in the execution and suppression of discrete and rhythmic movements. Sci Rep 11(1):22364.

Iwama S, Tsuchimoto S, Hayashi M, Mizuguchi N, Ushiba J (2020) Scalp electroencephalograms over ipsilateral sensorimotor cortex reflect contraction patterns of unilateral finger muscles. Neuroimage 222:117249.

Kang L, Ranft J, Hakim V (2023) Beta oscillations and waves in motor cortex can be accounted for by the interplay of spatially structured connectivity and fluctuating inputs. Elife 12:e81446.

Long Z, Fu Q, Fu X (2024) How mind wandering influences motor control: The modulating role of movement difficulty. NeuroImage 294:120638.

Merrick CM, Dixon TC, Breska A, Lin J, Chang EF, King-Stephens D, Laxer KD, Weber PB, Carmena J, Knight RT, Ivry RB. Left hemisphere dominance for bilateral kinematic encoding in the human brain. Elife. 2022 Mar 1;11:e69977.

Marques LM, Barbosa SP, Pacheco-Barrios K, Goncalves FT, Imamura M, Battistella LR, Simis M, Fregni F (2022) Motor event-related synchronization as an inhibitory biomarker of pain severity, sensitivity, and chronicity in patients with knee osteoarthritis. Neurophysiol Clin 52:413-26.

Rothwell J, Antal A, Burke D, Carlsen A, Georgiev D, Jahanshahi M, Sternad D, Valls-Solé J, Ziemann U (2021) Central nervous system physiology. Clin Neurophysiol 132:3043-3083.

Takasawa E, Abe M, Chikuda H, Hanakawa T (2022) A computational model based on corticospinal functional MRI revealed asymmetrically organized motor corticospinal networks in humans. Commun Biol 5:664.

Säisänen L, Könönen M, Niskanen E, Lakka T, Lintu N, Vanninen R, Julkunen P, Määttä S (2021) Primary hand motor representation areas in healthy children, preadolescents, adolescents, and adults. Neuroimage 228:117702.

Wischnewski M, Haigh ZJ, Shirinpour S, Alekseichuk I, Opitz A (2022) The phase of sensorimotor mu and beta oscillations has the opposite effect on corticospinal excitability. Brain Stimul 15:1093-100." ----------------------------------------------------------------------------------------------------------------- TMS Methodology:

The TMS protocol used to assess IHI is appropriate, but further elaboration on how stimulation intensities were chosen and their potential variability across subjects would help clarify the robustness of the results. Were intensities adjusted based on individual motor thresholds or were standard protocols used? Response:

We sincerely appreciate the reviewer's insightful comment regarding our TMS methodology. In response, we have made several modifications to the methodology section to enhance clarity and detail. These changes include: "The evaluation of IHI was conducted in accordance with the protocol developed by Talelli et al. (2008) and Zhang et al. (2024). The stimulation intensity for both the CS and TS was adjusted to evoke MEPs ranging from 1 to 1.5 mV in the contralateral FDI muscle at rest. The stimulation intensities of the CS and TS were maintained at consistent levels, with panel intensities set at the same value, and the adjustment range was between 110% and 120% of the RMT. Specifically, the TS intensity was set at 115% {plus minus} 5% of RMT, while the CS intensity was set at 114% {plus minus} 5% of RMT. Furthermore, when adjusting the TS intensity, it was imperative to ensure that in 10 consecutive stimulations, at least 5 MEP values in the right-hand FDI muscle fell between 1.5 and 2 mV to maintain consistent TS levels across different conditions" (Page 14-15) ----------------------------------------------------------------------------------------------------------------- Cohesion between Methods and Results:

While the methods are well-described, the results section feels slightly dense. Consider streamlining some of the descriptions of statistical tests to enhance readability. It might be helpful to include summary tables for the most important statistical outcomes, making it easier for readers to quickly grasp key findings.

Response:

We sincerely appreciate your thoughtful feedback regarding the presentation of our results. In response, we have made the following adjustments:

We acknowledge that the figures displaying our results may not have been clearly marked in the text. To address this, we have made corrections to enhance their distinguishability: "As illustrated in Fig. 4, the coherence between CM1 and IM1 was quantified. There was no significant difference in coherence between ballistic and self-paced movements (F(1,14) = 0.149, p = 0.705). Therefore, baseline analyses were not further pursued. Significant effects of speed (ballistic, self-paced) (F(1,31) = 12.030, p = 0.005) and time course (MRBD period, PMBS period) (F(1,31) = 14.485, p = 0.003) were observed, as well as a significant effect of their interaction (F(1,31) = 7.788, p = 0.016) (Fig. 4C)" (Page 25-26) "During the MRBD period, a notable effect of coherence direction (CM1 to IM1, IM1 to CM1) was observed (F(1,31) = 11.220, p = 0.015) (Fig. 5C)" (Page 27) "During the PMBS period, the effects of directed coherence (CM1 to IM1, IM1 to CM1) were found to be significant (F(1,31) = 53.097, p < 0.001), as were the effects of speed (ballistic, self-paced) (F(1,31) = 7.707, p = 0.024) (Fig. 5G)" (Page 27) We have summarized our results in three tables, clearly marked within the text for easy reference. "The results of ANOVAs are presented in Table 1" (Page 28) Table 1. Results of the ANOVAs "The results of the correlation analysis conducted during the MRBD and PMBS periods are presented in Tables 2 and 3, respectively" (Page 30) Table 2. Results of the correlation during MRBD period Table 3. Results of the correlation during PMBS period To minimize redundancy, we have removed the non-significant results previously listed in the table from the text. The corrected text now includes: "A significant effect of hemisphere (CM1, IM1) was demonstrated for the MRBD value (Fig. 3C) (F(1,31) = 12.737, p = 0.003)" (Page 23) "For PMBS values (Fig. 3C), a significant effect of speed (ballistic, self-paced) (F(1,31) = 21.078, p < 0.001) and hemisphere (CM1, IM1) (F(1,31) = 14.064, p = 0.003) was observed" (Page 24) "As illustrated in Figure 4, the coherence between CM1 and IM1 was quantified. Significant effects of speed (ballistic, self-paced) (F(1,31) = 12.030, p = 0.005) and time course (MRBD period, PMBS period) (F(1,31) = 14.485, p = 0.003) were observed, as well as a significant effect of their interaction (F(1,31) = 7.788, p = 0.016)" (Page 25-26) "During the MRBD period, a notable effect of coherence direction (CM1 to IM1, IM1 to CM1) was observed (F(1,31) = 11.220, p = 0.015)" (Page 27) "During the PMBS period, the effects of directed coherence (CM1 to IM1, IM1 to CM1) were found to be significant (F(1,31) = 53.097, p < 0.001), as were the effects of speed (ballistic, self-paced) (F(1,31) = 7.707, p = 0.024)" (Page 27) "As illustrated in Fig. 6ABC, no correlations were identified between speed and MRBD or hemispheric coupling. The directed coherence from CM1 to IM1 was not found to be dependent on speed during the MRBD period" (Page 28) "As illustrated in Fig. 7ABC, no correlation was identified between MRBD and coherence or directed coherence during the MRBD period" (Page 29) "As illustrated in Fig. 8AB, no notable impact of speed (ballistic, self-paced), the timing of TMS pulse initiation (PMBS peak time, PMBS recovery baseline time), or their interaction on TS MEP was discerned" (Page 30) "As illustrated in Fig. 9, the analysis revealed that there were significant effects of speed (ballistic, self-paced) (F(1,31) = 16.406, p = 0.005), timing of TMS triggering (PMBS peak time, PMBS recovery baseline time) (F(1,31) = 73.142, p < 0.001), and their interaction (F(1,31) = 16.415, p = 0.005) on IHI" (Page 31) We have also removed the term 'ANOVA' in brackets following the F and p values, as it does not provide additional clarity in this context.

----------------------------------------------------------------------------------------------------------------- Potential Applications:

The practical applications of these findings in motor rehabilitation or brain-computer interfaces should be highlighted more clearly. Could enhancing interhemispheric coupling through targeted stimulation improve motor recovery post-stroke? Response:

We sincerely appreciate the reviewer's insightful suggestion to highlight the practical applications of our findings. In response, we have added several lines to the conclusion section that address potential applications in motor rehabilitation and brain-computer interfaces. "The present study demonstrated that speed not only exerts control over PMBS but also influences interhemispheric functional connectivity, thereby regulating IHI during the PMBS period. The occurrence of ballistic movements has been observed to result in increased suppression from IM1 to CM1, thereby underscoring the pivotal role of the ipsilateral hemisphere in motor control. The present study elucidates the excitatory and inhibitory neurophysiological mechanisms of PMBS and its role in hemispheric information exchange. It is therefore proposed that PMBS may serve as a biomarker for movement states. The use of brain-computer interface (BCI) technology allows for the monitoring and regulation of PMBS, providing a clear avenue for the development of closed-loop, brain-controlled TMS stimulation systems with the potential to enhance stroke rehabilitation. Furthermore, it offers a neurophysiological foundation for the optimization of target stimulation areas in TMS interventions" (Page 39-40) ----------------------------------------------------------------------------------------------------------------- Statistics: The statistical analysis appears to be methodologically sound. The use of repeated-measures ANOVA and correlation analyses is appropriate, but the manuscript should provide more justification for the use of a linear model to describe the relationship between speed and neural metrics. It might be useful to explore non-linear models, as the relationships could be more complex.

Response:

We sincerely appreciate the thoughtful suggestion to explore non-linear models and fully acknowledge that such relationships may exist within our data. After careful consideration, we chose to employ a linear model for its simplicity and interpretability. To enhance the article's readability, we have added several lines to clarify our justification for this choice. "This study is an extension of our previous research, which employed a linear model to elucidate the relationships between neural data (Zhang et al., 2020; Zhang et al., 2024). In addition to the aforementioned studies, linear models have been demonstrated to offer a robust and straightforward methodology for elucidating correlations and causality in intricate datasets, while avoiding the pitfalls of overcomplicated analysis (Gourévitch et al., 2006; Ritchie et al., 2019; Penconek, 2022)" (Page 22) ----------------------------------------------------------------------------------------------------------------- Extended Data: Extended data sets, such as coherence and directed coherence analysis, are critical to this study. These should be included as they support the key results, especially regarding the differences in coherence across the MRBD and PMBS periods. However, a brief explanation of how coherence values were processed across subjects would enhance clarity.

Response:

We would like to express our sincere gratitude for your insightful comment regarding the inclusion of extended datasets and explanation of coherence and directed coherence analysis. And we have made corrections to improve clarity and readability throughout the manuscript: "This study is an extension of our previous research, which employed a linear model to elucidate the relationships between neural data (Zhang et al., 2020; Zhang et al., 2024). In addition to the aforementioned studies, linear models have been demonstrated to offer a robust and straightforward methodology for elucidating correlations and causality in intricate datasets, while avoiding the pitfalls of overcomplicated analysis (Gourévitch et al., 2006; Ritchie et al., 2019; Penconek, 2022)" (Page 8-9) We sincerely appreciate your insightful comment regarding the coherence analysis in our study. In response, we have added the following paragraph to the "Data Processing" subsection under "EEG Analysis": "In order to process coherence values across subjects, individual coherence values were initially calculated for each subject using the previously described method. Subsequently, the values were averaged across all subjects in order to obtain group-level coherence estimates" (Page 19) "To process the directed coherence values across subjects, the individual directed coherence values were initially calculated for each subject using the previously described method. Subsequently, the values were averaged across all subjects in order to obtain group-level estimates" (Page 21) ----------------------------------------------------------------------------------------------------------------- Software: The manuscript mentions the use of EEGLab for ICA and Spike2 for EMG analysis. While these are standard, a brief description of any custom code or pre-processing scripts used would be helpful. If possible, sharing the code would further support reproducibilit.

Response:

We sincerely appreciate your thoughtful suggestion regarding the software and code used in our study. We have uploaded all relevant custom code and pre-processing scripts to a public GitHub repository, accessible at https://github.com/Morinooku/code-for-eN-TNWR-0370-24X-.git. This repository includes custom MATLAB scripts for EEG processing. Please note that we did not develop custom code for EMG processing, as we utilized the standard functions provided by Spike2.

To further enhance the clarity of our methodology, we have added a sentence to the manuscript that states: "Code Availability The code for this article can be accessed online at: https://github.com/Morinooku/code-for-eN-TNWR-0370-24X-.git" (Page 40) Furthermore, We sincerely apologize for the oversight in not mentioning the use of the Fieldtrip and EEGlab toolboxes for EEG data processing. We have now included the following sentence in the "EEG Analysis" section of the "data processing" segment: "EEG was analyzed using the Matlab-based FieldTrip toolbox (Matlab, 2021b), developed by the Donders Institute for Brain, Cognition, and Behavior (http://www.ru.nl/neuroimaging/fieldtrip/), and the EEGLAB toolbox (Delorme and Makeig, 2004), developed by the Swartz Center for Computational Neuroscience (https://sccn.ucsd.edu/eeglab/)" (Page 15) ----------------------------------------------------------------------------------------------------------------- Sample Size and Statistical Power To ensure the credibility of the results, the authors should provide a statistical power calculation to demonstrate whether the current sample size is adequate to answer the research questions posed. This would help alleviate concerns about potential bias due to the limited number of subjects.

Response:

We would like to sincerely thank the editor and reviewers for their insightful comments regarding sample size and statistical power. And we have made some corrections to enhance clarity: "The present study is a preliminary, small-sample investigation. The sample size was determined based on previous studies employing similar neurophysiological techniques (Stancák and Pfurtscheller, 1996; Leocani et al., 1997; Manganotti et al., 1998; Mima et al., 2000; Serrien and Brown, 2002; Toma et al., 2002; Szurhaj et al., 2003; Gross et al., 2005; Yao et al., 2007; Stevenson et al., 2011; Qiu et al., 2016; Little et al., 2019). The mean number of participants in these studies was approximately 7.33. We initially recruited 10 volunteers; however, two withdrew due to personal circumstances. Ultimately, eight subjects completed the experiment, which exceeds the average sample size of the aforementioned studies" (Page 8-9) ----------------------------------------------------------------------------------------------------------------- Beta Power During the MRBD Phase The authors should conduct a control analysis to ensure there is sufficient power above zero during the MRBD phase, thus validating the coherence and directed coherence analyses between regions during this period.

Response:

We sincerely appreciate the valuable comment regarding the potential issue of reduced beta power during the MRBD phase and its impact on the coherence and directed coherence analyses between regions. MRBD refers to a ratio rather than an absolute value. MRBD is neurally meaningful as long as there is a noticeable decrease in beta power during the time window, regardless of whether the value is above zero. We have also made the following corrections: "The EEG power at each frequency and time point was normalized by subtracting the average power during the baseline period and then dividing it by the average baseline power. The resulting value was then multiplied by 100, thus expressing the event-related EEG power change relative to the baseline. The aforementioned procedure is described in detail by Nakayashiki et al. (2014).:

P_rest represents the mean power spectrum during the rest period (T_rest) and relative power at each time point and the relative power at each time point (RP(n)) was calculated using the instantaneous power spectrum at each time point (P_n)" (Page 16) ----------------------------------------------------------------------------------------------------------------- Baseline Period Analysis A baseline period control analysis should be included. This would confirm that no significant effects of beta power or coherence are present within the baseline period, ensuring that the measured effects are specific to the post-movement time points. Given the block design of this study, where trials are not interleaved, this is especially crucial for the robustness of the findings.

Response:

We sincerely appreciate the insightful suggestion regarding the inclusion of a baseline control analysis. Additional analyses revealed no significant differences between-group of different speeds in preparatory coherence levels. Therefore, we added the following lines to our text, and baseline discussion was not further considered: "There was no significant difference in coherence between ballistic and self-paced movements (F(1,14) = 0.149, p = 0.705). Therefore, baseline analyses were not further pursued" (Page 25) ----------------------------------------------------------------------------------------------------------------- Terminology Consistence There were inconsistencies in terminology throughout the manuscript. Terms such as "an increase in ERSP" should be clarified as "an increase in spectral power," and ERS and PMBS should not be used interchangeably. Cleaning up the terminology will greatly improve the manuscript's clarity and readability.

Response:

We sincerely thank the reviewer for highlighting the inconsistencies in terminology throughout our manuscript. To address this, we have replaced "ERD" and "ERS" with "MRBD" and "PMBS" where appropriate, ensuring consistency. "ERSP" is now only used in the introduction. These revisions help streamline the terminology and enhance clarity. The following corrections have also been made:

Changing "Event-related spectral perturbation (ERSP), including bilateral movement-related beta desynchronization (MRBD) and post-movement beta synchronization (PMBS)" to "Beta event-related spectral perturbation (ERSP), including bilateral movement-related beta desynchronization (MRBD) and post-movement beta synchronization (PMBS)" (Page 3) Changing " Reflected by electroencephalography (EEG) signals, this phenomenon is represented by an event-related spectral perturbation (ERSP) " to "Reflected by electroencephalography (EEG) signals, this phenomenon is represented by an beta event-related spectral perturbation (ERSP)" (Page 5) Changing "ERSP encompasses a decreased power change relative to the baseline, namely movement related beta desynchronization (MRBD), followed by an increase in ERSP after the termination of movement, known as post-movement beta synchronization (PMBS)" to "Beta ERSP encompasses a decreased beta power change relative to the baseline, namely movement related beta desynchronization (MRBD), followed by an increase in beta power after the termination of movement, known as post-movement beta synchronization (PMBS)" (Page 5) Changing "The association between coherence and ERSP over different time courses has been investigated in previous studies" to "The association between coherence and MRBD and PMBS over different time courses has been investigated in previous studies" (Page 5) Changing "Leocani et al. reported an increase in coherence over sensorimotor and frontal areas during the ERD phase" to "Leocani et al. (1997) reported an increase in coherence over sensorimotor and frontal areas during the MRBD phase" (Page 5) Changing "However, increasing evidence suggests that the motor cortex does not remain in an idle state during the beta ERS period but is involved in motor modulation" to "However, increasing evidence suggests that the motor cortex does not remain in an idle state during the PMBS period but is involved in motor modulation" (Page 6) Changing "An increase in beta rhythm power (i.e., PMBS) could be observed after the isometric abduction movement" to "PMBS could be observed after the isometric abduction movement" (Page 11) Changing "The ERSP waveforms, which include two stages: MRBD during the execution of the motion and PMBS after movement while performing the task" to "The beta waveforms, which include two stages: MRBD during the execution of the motion and PMBS after movement while performing the task" (Page 12) Changing "The latency of beta rebound was identified as the time at which beta power increased by more than 10% above baseline" to "The latency of PMBS was identified as the time at which beta power increased by more than 10% above baseline" (Page 17) Changing "Repeated measures two-way ANOVAs were also conducted to study the effect of speed (ballistic, self-paced) and time course of ERSP (MRBD period, PMBS period) on interhemispheric coherence and interhemispheric directed coherence" to "Repeated measures two-way ANOVAs were also conducted to study the effect of speed (ballistic, self-paced) and time course (MRBD period, PMBS period) on interhemispheric coherence and interhemispheric directed coherence" (Page 21) Changing "Repeated-measures ANOVA showed a significant effect of speed (ballistic, self-paced) (F(1,31) = 12.030, p = 0.005, ANOVA) and time course of ERSP (MRBD period, PMBS period) (F(1,31) = 14.485, p = 0.003, ANOVA) with a significant effect of their interaction(F(1,31) = 7.788, p = 0.016, ANOVA)" to "As illustrated in Fig. 4, the coherence between CM1 and IM1 was quantified. Significant effects of speed (ballistic, self-paced) (F(1,31) = 12.030, p = 0.005) and time course (MRBD period, PMBS period) (F(1,31) = 14.485, p = 0.003) were observed, as well as a significant effect of their interaction (F(1,31) = 7.788, p = 0.016)" (Page 25-26) Changing "Directed coherence at different time points of ERSP" to "Directed coherence at different time points of MRBD and PMBS" (Page 26) Chnaging "Fig. 5 presents the results of directed coherence during different time courses of ERSP" to "Fig. 5 presents the results of directed coherence during different time courses of MRND and PMBS" (Page 27) ----------------------------------------------------------------------------------------------------------------- Data Availability Regarding the data availability statement, and given that the data can be anonymized, the authors should make the data publicly available, which aligns with open science principles and could enhance the reproducibility of their work.

Response:

We sincerely thank the reviewer for the valuable suggestion regarding our data availability statement and for emphasizing the importance of open science principles. As mentioned earlier (response to the sixth comment of the first reviewer), the participants did not explicitly consent to broad data distribution. Additionally, the data are currently being used in ongoing studies awaiting publication, and releasing them prematurely may affect the novelty of these works. After all related research is published and participant consent is obtained, we will be pleased to make the data available.

We believe this approach balances our ethical obligations, preserves the integrity of our ongoing work, and aligns with the principles of openness and reproducibility in scientific research. Thank you again for your invaluable feedback, which has helped us improve the impact and accessibility of our research.

----------------------------------------------------------------------------------------------------------------- TMS Methodology and Stimulation Intensity The authors should provide more details on how the stimulation intensities were chosen and discuss the potential variability across subjects. Additional details in this area would help clarify the robustness of the results.

Response:

We sincerely thank the reviewer for raising this important point regarding our TMS methodology and stimulation intensity. In response, we have revised the methodology section to include the following details: "The evaluation of IHI was conducted in accordance with the protocol developed by Talelli et al. (2008) and Zhang et al. (2024). The stimulation intensity for both the CS and TS was adjusted to evoke MEPs ranging from 1 to 1.5 mV in the contralateral FDI muscle at rest. The stimulation intensities of the CS and TS were maintained at consistent levels, with panel intensities set at the same value, and the adjustment range was between 110% and 120% of the RMT. Specifically, the TS intensity was set at 115% {plus minus} 5% of RMT, while the CS intensity was set at 114% {plus minus} 5% of RMT. Furthermore, when adjusting the TS intensity, it was imperative to ensure that in 10 consecutive stimulations, at least 5 MEP values in the right-hand FDI muscle fell between 1.5 and 2 mV to maintain consistent TS levels across different conditions" (Page 14-15) ----------------------------------------------------------------------------------------------------------------- Relationship Between PMBS and Movement Speed The results showing stronger coherence during ballistic movements in the PMBS phase are intriguing. The authors should delve deeper into the reasons for this discrepancy, potentially exploring factors like task complexity or difficulty.

Response:

We sincerely thank the editor and reviewers for their insightful comment on exploring task complexity and movement speed as factors contributing to the stronger coherence observed during ballistic movements in the PMBS phase. In response, we have conducted a more detailed analysis to address this discrepancy. "An increase in task complexity is associated with enhanced inter-cerebral coherence (Gross et al., 2005; Aliakbaryhosseinabadi et al., 2021), which may explain the observed discrepancies. In contrast to studies investigating simple, self-paced movements (Andrew and Pfurtscheller, 1999; Leocani et al., 1997), we introduced more constrained and precisely timed tasks that demand higher motor control and coordination. These more challenging tasks may prompt the brain to exceed the threshold for interhemispheric interaction compared to simpler tasks. Another critical factor in motor cortical dynamics is movement speed. Previous studies primarily focused on self-paced movements without controlling for speed (Andrew and Pfurtscheller, 1999; Leocani et al., 1997). In contrast, our study tested two distinct conditions: ballistic (~130 ms) and self-paced (~180 ms). This highlights the pivotal role of speed in interhemispheric interactions, with earlier studies potentially lacking coherence due to their exclusive focus on self-paced movements (Gross et al., 2005; Andrew and Pfurtscheller, 1999; Leocani et al., 1997)" (Page 37) The references mentioned are available in the article's reference section, except for Feingold et al. (2015), Fronso et al. (2018), and Keinrath et al. (2006). These articles are listed below:

Feingold J, Gibson DJ, DePasquale B, Graybiel AM (2015) Bursts of beta oscillation differentiate postperformance activity in the striatum and motor cortex of monkeys performing movement tasks. Proc Natl Acad Sci 112:13687-13692.

Fronso S, Tamburro G, Robazza C, Bortoli L, Comani S, Bertollo M (2018) Focusing attention on muscle exertion increases EEG coherence in an endurance cycling task. Front Psychol 9:1249.

Keinrath C, Wriessnegger S, Müller-Putz GR, Pfurtscheller G (2006) Post-movement beta synchronization after kinesthetic illusion, active and passive movements. Int J Psychophysiol 62:321-327.
